# Endothelial Dicer promotes atherosclerosis and vascular inflammation by miRNA-103-mediated suppression of KLF4

**DOI:** 10.1038/ncomms10521

**Published:** 2016-02-03

**Authors:** Petra Hartmann, Zhe Zhou, Lucia Natarelli, Yuanyuan Wei, Maliheh Nazari-Jahantigh, Mengyu Zhu, Jochen Grommes, Sabine Steffens, Christian Weber, Andreas Schober

**Affiliations:** 1 Institute for Cardiovascular Prevention, Ludwig-Maximilians University Munich, Pettenkoferstrasse 9, 80336 Munich, Germany; 2 Institute for Molecular Cardiovascular Research, RWTH Aachen University, Pauwelsstrasse 30, 52074 Aachen, Germany; 3 DZHK (German Centre for Cardiovascular Research), Partner Site Munich Heart Alliance, Biedersteiner Strasse 29, 80802 Munich, Germany; 4 European Vascular Center Aachen-Maastricht, Medical University Maastricht, P. Debyelaan 25, 6229 HX Maastricht, The Netherlands; 5 European Vascular Center Aachen-Maastricht, RWTH Aachen University, Pauwelsstrasse 30, 52074 Aachen, Germany

## Abstract

MicroRNAs regulate the maladaptation of endothelial cells (ECs) to naturally occurring disturbed blood flow at arterial bifurcations resulting in arterial inflammation and atherosclerosis in response to hyperlipidemic stress. Here, we show that reduced endothelial expression of the RNAse Dicer, which generates almost all mature miRNAs, decreases monocyte adhesion, endothelial C–X–C motif chemokine 1 (CXCL1) expression, atherosclerosis and the lesional macrophage content in apolipoprotein E knockout mice (*Apoe*^−/−^) after exposure to a high-fat diet. Endothelial Dicer deficiency reduces the expression of unstable miRNAs, such as miR-103, and promotes Krüppel-like factor 4 (KLF4)-dependent gene expression in murine atherosclerotic arteries. MiR-103 mediated suppression of KLF4 increases monocyte adhesion to ECs by enhancing nuclear factor-κB-dependent CXCL1 expression. Inhibiting the interaction between miR-103 and KLF4 reduces atherosclerosis, lesional macrophage accumulation and endothelial CXCL1 expression. Overall, our study suggests that Dicer promotes endothelial maladaptation and atherosclerosis in part by miR-103-mediated suppression of KLF4.

## Abstract (web summary)

The RNAse III endonuclease Dicer is crucial for processing of pre-miRNAs in health and disease. Here the authors show that endothelial Dicer promotes atherosclerosis by increasing miR-103 levels leading to suppression of the anti-inflammatory transcription factor KLF4, thus suggesting a novel approach to treat this disease.

## Graphical abstract

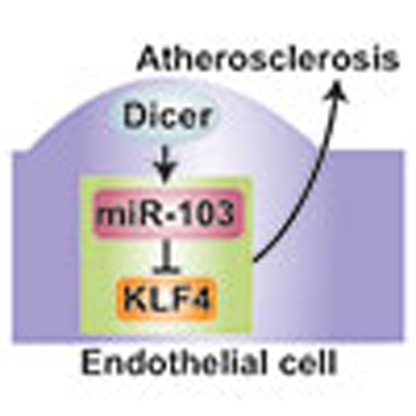

Endothelial cells (ECs) are perfectly adapted to conduct blood through high-shear stress, high-pressure environment in unbranched segments of arteries^1^. High-shear stress at the endothelial surface induces the transcription factors Krüppel-like factors (KLF) 2 and KLF4, which promote a quiescent EC phenotype characterized by a low turnover rate, tight intercellular junctions with low permeability, reduced inflammatory activation and antithrombotic properties^2^,^3^,^4^. However, distribution of blood throughout the body requires branching of arteries where blood flow is naturally disturbed and consequently shear stress is low^5^. Disturbed flow at arterial bifurcations constantly damages the endothelium by activating the endoplasmic reticulum stress response, suppresses EC-specific transcriptional programmes by downregulating KLF2/4, and increases the activity of the proinflammatory transcription factor nuclear factor-κB (NF-κB)^6^,^7^,^8^. Moreover, the deposition of chemically modified lipoproteins in the subendothelial space aggravates the maladaptive response of ECs and results in the accumulation of macrophages derived from circulating monocytes in the arterial wall during atherosclerosis^9^,^10^,^11^. Inflammatory activation of macrophages triggers the secretion of inflammatory cytokines, such as tumour necrosis factor-α (TNF-α) and interleukin-1, which further promotes endothelial maladaptation by activating NF-κB^12^,^13^. Endothelial chemokines, such as chemokine (C–X–C motif) ligand 1 (CXCL1), chemokine (C–C motif) ligand 2 (CCL2) and chemokine (C–X3–C) ligand 1 (CX_3_CL1), have key roles in the accumulation of macrophages in atherosclerotic lesions^14^,^15^,^16^,^17^. ECs secrete CXCL1 and CCL2 from intracellular storage compartments upon activation by thrombin or lipoprotein oxidation products^15^,^18^. Whereas CXCL1 is immobilized on the endothelial surface and triggers monocyte adhesion by activating integrins on the monocyte surface^15^,^19^, CCL2 contributes to macrophage infiltration probably by regulating hypercholesterolemia-induced monocytosis^17^,^20^. The membrane-bound chemokine CX_3_CL1 is upregulated in inflamed ECs and promotes atherogenic monocyte adhesion by activating platelets^21^. Constitutive and induced expression of CXCL1, CCL2 and CX_3_CL1 are controlled by the activity of NF-κB^22^,^23^.

MicroRNAs (miRNAs) are small noncoding RNAs of ∼22 nucleotides that regulate a number of processes related to atherogenesis, such as macrophage activation and the phenotype of vascular smooth muscle cells (SMCs), by translational repression or degradation of their target mRNAs^24^,^25^,^26^. Mature miRNA sequences are embedded in the stem-loop structure of their primary miRNA (pri-miRNA) transcripts. The nuclear RNAse III Drosha crops the pri-miRNA stem at the 5′ and 3′ sides to release a ∼65-nucleotide-long, hairpin-shaped precursor miRNA (pre-miRNA). In the cytoplasm, the RNAse III endonuclease Dicer cleaves all pre-miRNAs, except pre-miR-451, near the terminal loop of the hairpin into 21 to 25-nucleotide-long miRNA duplexes, which are subsequently loaded onto Argonaut proteins^27^. Although one strand of the miRNA duplex is selected during the loading step to generate the RNA-induced silencing complex (RISC), the second strand is usually removed and degraded. Binding to Argonaut proteins protects miRNAs from degradation by exonucleases and thus greatly increases their stability compared with that of mRNAs. However, the expression level of a small subset of miRNAs is highly dependent on continuous biogenesis because of a fast turnover rate^28^,^29^.

Deletion of the gene encoding Dicer in mice compromises blood vessel formation and causes embryonic lethality, indicating an essential role of this endonuclease in vertebrate development^30^,^31^. In adults, Dicer is essential for the function of various cell types, such as pancreatic beta cells and cardiomyocytes^32^,^33^, and reduced Dicer expression contributes to aging and promotes cancer development^34^,^35^. Although deletion of Dicer in SMCs results in embryonic lethality^36^, mice with deficiency of Dicer in ECs develop normally and have no overt phenotype^37^. However, reduced endothelial Dicer expression severely impairs postnatal angiogenesis and limits the proliferation and migration of ECs^37^,^38^,^39^. Notably, knockdown of Dicer induces upregulation of EC-specific genes, such as endothelial nitric oxide synthase (eNOS), Angiopoietin-1 receptor (Tie2) and vascular endothelial growth factor receptor 2 (KDR), indicating that Dicer generates miRNAs that impair endothelial differentiation^38^. Disturbed blood flow increases the expression of several endothelial miRNAs, including miR-92a and miR-712, which promote atherosclerosis by targeting KLF2/4 and by increasing shedding of membrane-bound TNF-α, respectively^7^,^40^,^41^. Moreover, several miRNAs have been implicated in NF-κB pathway regulation in ECs. For example, miR-10a, miR-146a and miR-181b promote an anti-inflammatory phenotype in ECs by inhibiting the NF-κB pathway, whereas miR-19a has a proinflammatory role in ECs^42^,^43^,^44^,^45^. Although individual miRNAs affect endothelial inflammation, it is unclear which role Dicer-dependent miRNA biogenesis in ECs plays in atherosclerosis.

Here, we investigated the role of endothelial Dicer in monocyte adhesion and atherosclerosis. EC-specific deletion of Dicer in apolipoprotein E-knockout (*Apoe*^*−/−*^) mice reduced monocyte adhesion to the early atherosclerotic endothelium by downregulating CXCL1, resulting in diminished lesion formation. MiR-103 is downregulated in Dicer-deficient ECs and promoted CXCL1-mediated monocyte adhesion by targeting KLF4. Blocking the interaction between miR-103 and KLF4 in arteries reduced atherosclerosis, lesional macrophage accumulation and CXCL1 expression, similar as the deletion of Dicer in ECs. Overall, these data indicate that Dicer can enhance atherosclerosis and endothelial inflammation by increasing miR-103 expression.

## Results

### Endothelial Dicer regulates miRNAs during atherosclerosis

**Figure 1:**
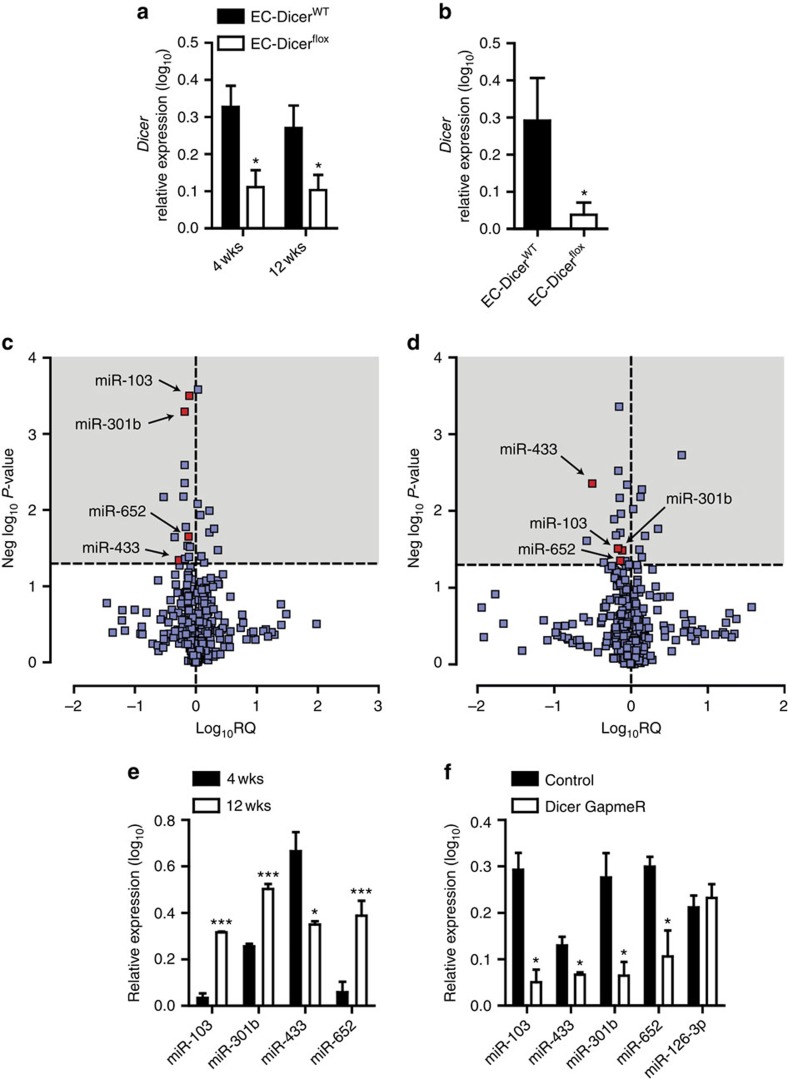
Effect of endothelial Dicer on miRNA expression during atherosclerosis. (**a**) Quantitative RT–PCR analyses of *Dicer* mRNA expression in the aortas from TMX-treated EC-Dicer^WT^ and EC-Dicer^flox^ mice fed a HFD for 4 or 12 weeks (wks; *n*=5 mice per group). (**b**) *Dicer* mRNA expression levels in aortic ECs isolated from EC-Dicer^WT^ and EC-Dicer^flox^ mice 2 weeks after TMX injection (*n*=3 per group). (**c**,**d**) Differentially expressed miRNAs (grey areas) in the aortas of EC-Dicer^flox^ mice compared with EC-Dicer^WT^ mice (*n*=3 mice per group) after exposure to a HFD for 4 (**c**) or 12 weeks (**d**). The expression profiles were determined using qRT–PCR arrays. RQ, relative quantification. (**e**) The expression levels of miR-103, miR-301b, miR-433 and miR-652 in the aortas of EC-Dicer^WT^ mice fed a HFD for 4 or 12 weeks (*n*=3 mice per group). (**f**) Quantitative RT–PCR analyses of miR-103, miR-301b, miR-433, miR-652 and miR-126-3p expression in human aortic ECs (HAECs) treated with Dicer-specific LNA-GapmeRs or non-targeting control LNA-GapmeRs (*n*=3–4 per group). The data are represented as the mean±s.e.m. of the indicated number (*n*) of repeats. *P<0.05; **P<0.01 and ***P<0.001 by Student's *t*-test.

Although many miRNAs are downregulated in atherosclerotic arteries^26^, *Dicer* expression in the aortas of *Apoe*^*−/−*^ mice was not affected by 12 weeks of high-fat diet (HFD) feeding compared with mice fed a normal diet (Supplementary Fig. 1a), indicating that miRNA biogenesis by Dicer is not generally impaired during early atherosclerosis. Moreover, the expression of *Dicer* in ECs was not affected by low-shear stress and did not differ between the aortic arch and thoracic aorta, indicating that Dicer is not regulated by blood flow (Supplementary Fig. 1b,c). To study the role of endothelial Dicer in atherosclerosis, we generated *Apoe*^*−/−*^ mice containing a loxP site-flanked *Dicer* sequence (Dicer^flox^) and a transgene with Tamoxifen (TMX) inducible Cre recombinase under control of the EC-specific VE-cadherin (Cdh5) promoter. TMX administration reduced aortic *Dicer* mRNA expression in EC-Dicer^flox^ mice by 66% and 58% compared with EC-Dicer^WT^ mice after 4 and 12 weeks of HFD feeding, respectively (Fig. 1a). In ECs isolated from the aortas of EC-Dicer^flox^ mice injected with TMX, *Dicer* mRNA expression was decreased by 87% compared with ECs isolated from EC-Dicer^WT^ mice (Fig. 1b), whereas the expression of *Dicer* was not affected in myeloid cells from EC-Dicer^flox^ mice (Supplementary Fig. 1d). These results indicate that TMX treatment of EC-Dicer^flox^ mice effectively reduced Dicer expression in ECs.

To determine the effect of endothelial Dicer deficiency on miRNA biogenesis during atherosclerosis, the miRNA expression profile was determined in aortas from HFD-fed EC-Dicer^flox^ and EC-Dicer^WT^ mice by quantitative real-time PCR (qRT–PCR) arrays. After 4 weeks of HFD feeding, 14 miRNAs were downregulated and 9 miRNAs were upregulated in EC-Dicer^flox^ mice compared with EC-Dicer^WT^ mice (Fig. 1c and Supplementary Table 1). After 12 weeks of HFD exposure, 18 miRNAs and 8 miRNAs were down- and upregulated in EC-Dicer^flox^ mice, respectively (Fig. 1d and Supplementary Table 2). Notably, the expression levels of miR-103, miR-301b, miR-433 and miR-652 were downregulated in EC-Dicer^flox^ mice at the 4- and 12-week time points. In contrast to miR-301b, endothelial Dicer deficiency reduced the expression levels of miR-103, miR-433 and miR-652 in both aortic arch and thoracic aorta (Supplementary Fig. 2), indicating that the effect of endothelial Dicer on these miRNAs is independent of disturbed flow. Moreover, the expression levels of miR-103, miR-652 and miR-301b in EC-Dicer^WT^ mice were higher at the 12-week time point than the 4-week time point (Fig. 1e). In human aortic ECs (HAECs), silencing of Dicer using GapmeRs diminished the expression levels of miR-103, -301b, -652 and -433, but not that of miR-126-3p, after 24 h (Fig. 1f and Supplementary Fig. 3). In summary, these results suggest that endothelial Dicer deficiency selectively lowers the expression levels of unstable miRNAs, most of which are upregulated during atherosclerosis.

### Endothelial Dicer promotes atherosclerosis

**Figure 2:**
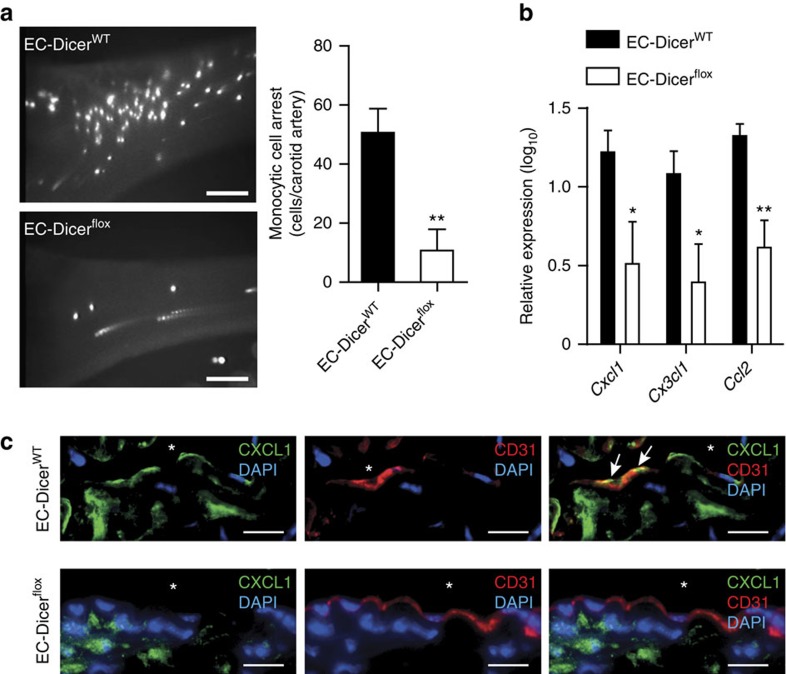
Role of endothelial Dicer in atherogenic monocyte adhesion. (**a**) *Ex vivo* perfusion assays showing monocytic cell arrest on the endothelia of the left carotid arteries of mice (*n*=4–5 mice per group) fed a HFD for 4 weeks. (**b**) Quantitative RT–PCR analyses of the mRNA expression levels of chemokines in carotid arteries of mice (*n*=3–4 mice per group) fed a HFD for 4 weeks. (**c**) Immunostaining of CXCL1 and the endothelial marker CD31 in carotid artery sections after 4 weeks of HFD feeding. Arrows indicate CXCL1 expressing ECs. Representative images of three independent experiments are shown. The nuclei were stained with 4',6-diamidino-2-phenylindole (DAPI). Scale bars, 30 μm (**a**), 12 μm (**c**). Asterisks indicate the lumen. The data are represented as the mean±s.e.m. of the indicated number (*n*) of repeats. *P<0.05 and **P<0.01 by Student's *t*-test.

**Figure 3:**
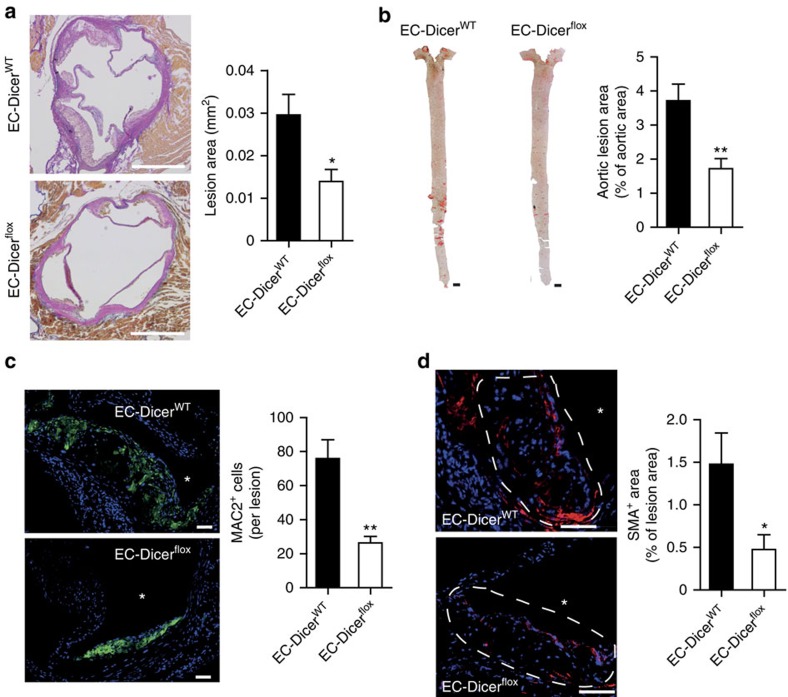
Loss of endothelial Dicer limits atherosclerosis. (**a**,**b**) Atherosclerotic lesion formation in mice fed a HFD for 12 weeks analysed in aortic root sections stained with elastic van Gieson stain (a; *n*=8 mice per group) and in *en face* prepared aortas stained with Oil red O stain (b; *n*=9–10 mice per group). (**c**,**d**) Macrophage and smooth muscle cell accumulation in aortic root lesions determined by immunostaining of MAC2 (**c**, green; *n*=7–9 mice per group) and smooth muscle actin (**d**, red; *n*=8 mice per group), respectively. The nuclei were counterstained with 4',6-diamidino-2-phenylindole (DAPI; blue). Scale bars, 500 μm (**a**), 50 μm (**c**,**d**) and 1 mm (**b**). Asterisks indicate the lumen. Dashed lines encircle atherosclerotic lesions. The data are represented as the mean±s.e.m. of the indicated number (*n*) of repeats. *P<0.05 and **P<0.01 by Student's *t*-test.

To determine the role of Dicer in endothelial inflammation, monocytic cell arrest was studied *ex vivo* using perfused carotid arteries from EC-Dicer^flox^ and EC-Dicer^WT^ mice that were fed a HFD for 4 weeks. Monocyte adhesion (Fig. 2a) and the expression levels of the *Cxcl1, Cx*_*3*_*cl1* and *Ccl2* mRNAs (Fig. 2b) were significantly lower in carotid arteries from EC-Dicer^flox^ mice than in those from EC-Dicer^WT^ mice. Reduced endothelial expression of CXCL1 in EC-Dicer^flox^ mice was identified by dual immunostaining of CXCL1 and the endothelial marker CD31 (Fig. 2c). These results suggest that endothelial Dicer enhances chemokine expression and may promote monocyte adhesion during the early stages of atherosclerosis. After 12 weeks of HFD feeding, atherosclerosis in the aortic roots (Fig. 3a) and thoracoabdominal aortas (Fig. 3b) of EC-Dicer^flox^ mice was 58% and 41% lower than that in EC-Dicer^WT^ mice, respectively. The distribution of atherosclerotic lesions in the aorta did not differ between EC-Dicer^flox^ and EC-Dicer^WT^ mice (Supplementary Fig. 4). The number of macrophages per lesion (Fig. 3c) and the SMC content (Fig. 3d) were diminished in EC-Dicer^flox^ mice. Deletion of the endothelial *Dicer* gene did not affect serum cholesterol levels (Supplementary Fig. 5). Taken together, these findings indicate that the expression of Dicer in ECs enhances atherosclerotic lesion formation.

### miR-103 induces endothelial inflammation

**Figure 4:**
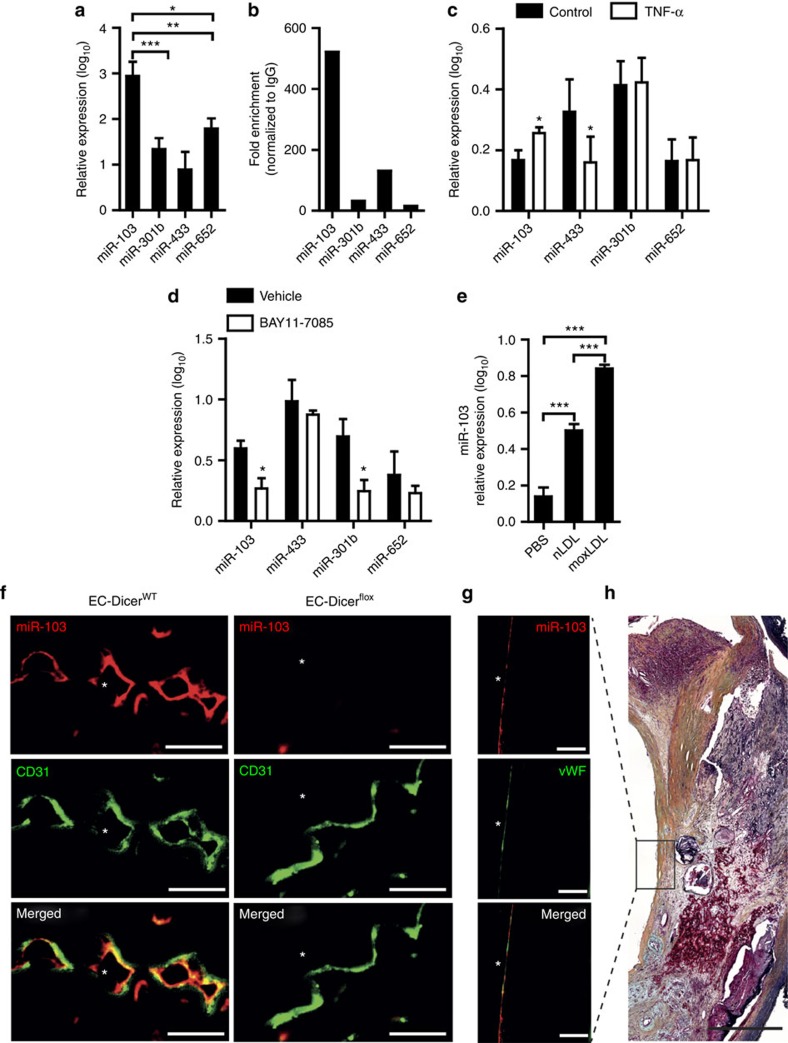
Expression of miR-103 in ECs during atherosclerosis. (**a**,**b**) The expression levels of miR-103, miR-301b, miR-433 and miR-652 (a; *n*=5 per group) and their enrichment in AGO2-IPs of human ECs (**b**). The results of **b** are expressed as the fold enrichment of miRNAs in AGO2-IP samples compared with control IgG-IP samples. Results of one representative experiment are shown in **b**. (**c**) Quantitative RT–PCR analyses of the expression levels of miR-103, miR-301b, miR-433 and miR-652 in HAECs with and without TNF-α stimulation (*n*=3–4 per group). (**d**) MiRNA expression level in HAECs treated with vehicle or the NF-κB-inhibitor BAY11-7085 (*n*=4–5 per group). (**e**) Expression levels of miR-103 in HAECs treated with PBS, native low-density lipoprotein (nLDL) or mildly oxidized-low-density lipoprotein (moxLDL; *n*=4 per group). (**f**) Combined *in situ* PCR detection of miR-103 and immunostaining of the endothelial marker CD31 in carotid sections from EC-Dicer^WT^ and EC-Dicer^flox^ mice fed a HFD for 4 weeks. Representative images of three independent experiments are shown. (**g**) Endothelial miR-103 expression in human atherosclerotic plaques determined by *in situ* PCR and immunostaining of von Willebrand factor (vWF). (**h**) Movat's pentachrome staining of a human atherosclerotic plaque section located adjacent to that used for the *in situ* detection of miR-103. The region of the plaque used for miR-103 expression analysis is indicated. Scale bars, 12 μm (**f**), 25 μm (**g**) and 500 μm (**h**). Asterisks indicate the lumen. The data are represented as the mean±s.e.m. of the indicated number (*n*) of repeats. *P<0.05, **P<0.01 and ***P<0.001 by Student's *t*-test and one-way analysis of variance.

Among the miRNAs downregulated in the aorta of EC-Dicer^flox^ mice, miR-103 was expressed most abundantly in human ECs (Fig. 4a) and was highly enriched in the RISCs of these cells, as determined using anti-argonaute 2 (AGO2)-immunoprecipitation, suggesting a prominent role for miR-103 in the regulation of EC function (Fig. 4b). Notably, the expression of miR-107, which shares the same seed sequence with miR-103, was not affected by endothelial *Dicer* deletion (Fig. 1c,d and Supplementary Tables 1 and 2). Stimulation of HAECs with TNF-α moderately induced miR-103 and suppressed miR-433 expression, whereas blocking of NF-κB reduced only the expression of miR-103 and -301b (Fig. 4c,d). In addition, the expression of miR-103 was upregulated in HAECs upon stimulation with native low-density lipoprotein (LDL) and further increased by mildly oxidized LDL treatment (Fig. 4e). These data suggest that NF-κB activity and hyperlipidemia drive the expression of miR-103 in atherosclerotic ECs, which may in turn indirectly regulate the chemokine expression.

In addition, miR-103 was highly expressed in the aortic endothelium after 4 and 12 weeks of HFD feeding in EC-Dicer^WT^ mice, whereas endothelial miR-103 expression was not detectable by *in situ* PCR in EC-Dicer^flox^ mice (Fig. 4f and Supplementary Fig. 6). Similarly, combined *in situ* PCR and immunostaining of von Willebrand factor (vWF) revealed prominent miR-103 expression in ECs covering human atherosclerotic lesions (Fig. 4g,h). These results suggest that Dicer-mediated generation of endothelial miRNAs, in particular miR-103, may play a crucial role in lesion formation.

**Figure 5:**
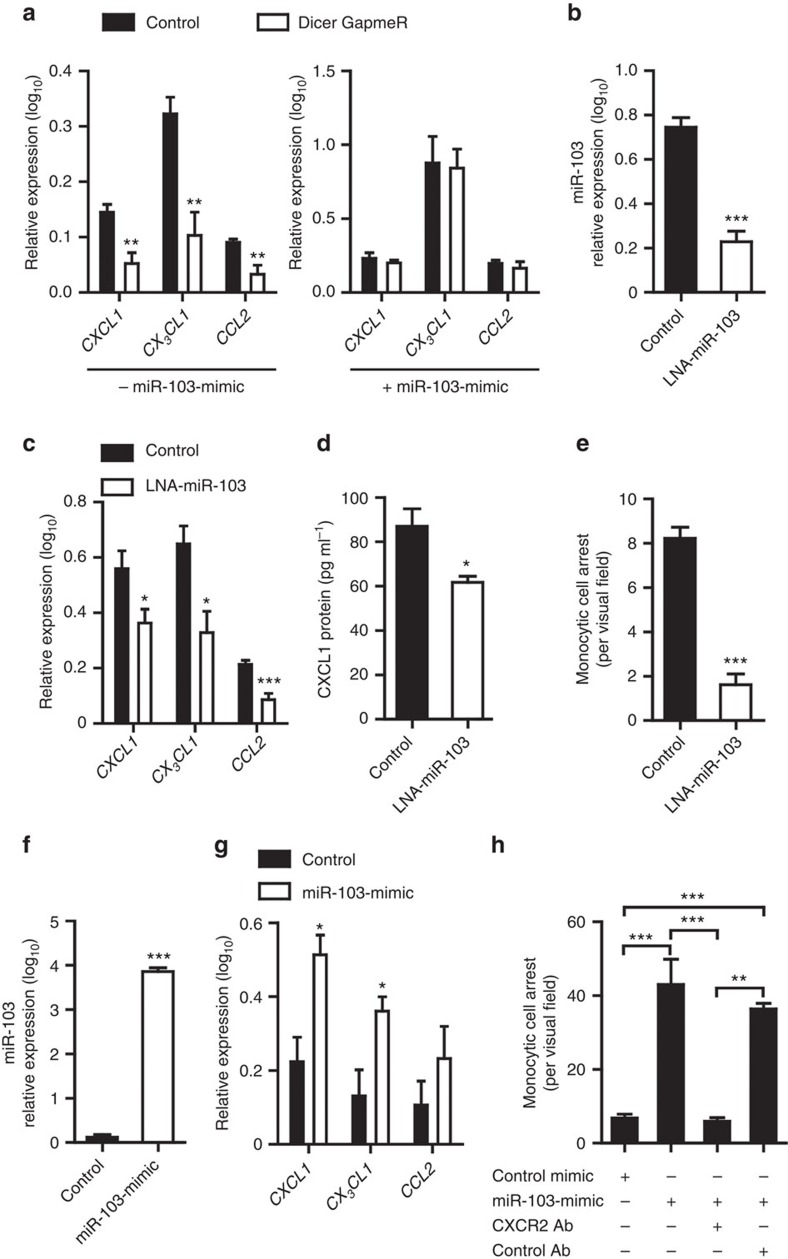
Effects of miR-103 on chemokine expression in ECs and monocyte adhesion. (**a**) Chemokine mRNA expression in HAECs treated with Dicer-specific LNA-GapmeRs or control LNA-GapmeRs with (right) or without (left) miR-103-mimic treatment (*n*=4–6 per group). (**b**,**c**) Quantitative RT–PCR analyses of miR-103 (**b**) and chemokine mRNA (**c**) expression levels in HAECs (*n*=5–6 per group) treated with LNA-inhibitors of miR-103 or non-targeting LNA-oligonucleotides. (**d**) ELISA of CXCL1 protein expression in HAEC lysates (*n*=3–4 per group) with and without miR-103 inhibition. (**e**) Flow chamber assays to determine monocyte adhesion to HAECs treated with LNA-inhibitors of miR-103 or control oligonucleotides (*n*=3 per group). (**f**,**g**) The expression of miR-103 (**f**) or chemokine mRNAs (**g**) in HAECs treated with miR-103-specific or negative control mimics (*n*=3–4 per group). (**h**) Adhesion of monocytes to HAECs treated with miR-103-mimics or control oligonucleotides under flow conditions. Monocytic cells were pretreated with or without an antibody to block CXCR2 or non-targeting control IgG (*n*=3 per group). The data are represented as the mean±s.e.m. of the indicated number (*n*) of repeats. *P<0.05, **P<0.01 and ***P<0.001 by Student's *t*-test (**b**–**d**) and one-way analysis of variance (**a**,**e**).

Overexpression of miR-103 was sufficient to upregulate the expression levels of the *CXCL1*, *CX*_*3*_*CL1* and *CCL2* mRNAs after silencing Dicer in HAECs (Fig. 5a). Furthermore, treatment of HAECs with a specific locked nucleic acid (LNA)-inhibitor, which reduced miR-103 expression by 70% (Fig. 5b), also reduced the expression of the *CXCL1*, *CX*_*3*_*CL1* and *CCL2* mRNAs significantly (Fig. 5c). Downregulation of CXCL1 was also confirmed at the protein level (Fig. 5d). Next, *in vitro* flow chamber assays were used to examine the effect of miR-103 on monocyte adhesion to ECs. Unlike non-targeting LNA-oligonucleotides, LNA-inhibitors of miR-103 attenuated the adhesion of monocytic cells to HAECs (Fig. 5e). Conversely, transfection of HAECs with miR-103 mimics, which increased miR-103 expression by 22-fold (Fig. 5f), upregulated the expression levels of *CXCL1* and *CX*_*3*_*CL1*, but had only a slight effect on the expression of *CCL2* (Fig. 5g). Furthermore, overexpression of miR-103 in HAECs increased monocytic cell adhesion, and this effect was reversed by blocking the CXCL1 receptor C–X–C chemokine receptor type 2 on monocytes (Fig. 5h). These findings suggest that reduced expression of miR-103 in EC-Dicer^flox^ mice results in diminished adhesion of monocytes to the carotid arteries.

### Endothelial Dicer regulates KLF4-dependent gene expression

**Figure 6:**
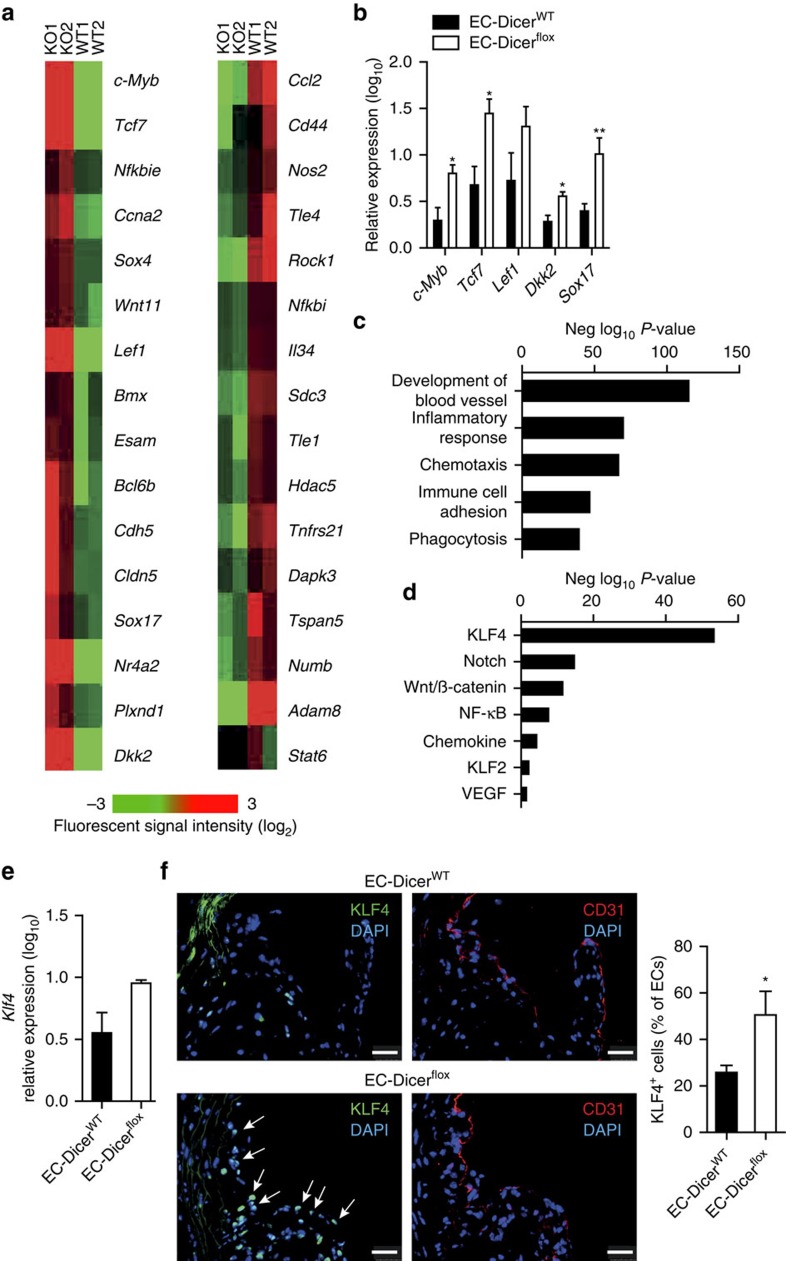
Effect of endothelial Dicer deletion on arterial gene expression. (**a**) Heat map of genes differentially expressed in the aortas of EC-Dicer^flox^ mice compared with EC-Dicer^WT^ mice (*n*=2 mice per group; *P*<0.05; fold change cutoff=1.2) after 12 weeks HFD feeding. (**b**) Quantitative RT–PCR analyses of the *c-Myb, Tcf7, Lef1, Dkk2* and *Sox17* expression levels in the aortas of EC-Dicer^WT^ and EC-Dicer^flox^ mice after 12 weeks HFD feeding (*n*=4–7 mice per group). (**c**,**d**) Significant enriched biological processes (**c**) and signalling pathways (**d**) among the genes differentially regulated in EC-Dicer^flox^ mice as compared with EC-Dicer^WT^ mice using Ingenuity Pathway Analysis software. (**e**) Quantitative RT–PCR analyses of *Klf4* mRNA expression in the aortas of EC-Dicer^WT^ and EC-Dicer^flox^ mice after 12 weeks of HFD feeding (*n*=4 mice per group). (**f**) KLF4^+^ ECs in aortic root sections of EC-Dicer^WT^ and EC-Dicer^flox^ mice identified by immunostaining of KLF4 and the endothelial marker CD31. The arrows indicate ECs with nuclear KLF4 staining. The nuclei were counterstained with 4',6-diamidino-2-phenylindole (DAPI). Representative images are shown (*n*=3–4 mice per group). Scale bar, 25 μm. The data are represented as the mean±s.e.m. of the indicated number (*n*) of repeats. *P<0.05; **P<0.01 by Student's *t*-test.

To study the mechanism by which deficiency of endothelial Dicer reduced lesion formation, genome-wide microarray analyses of atherosclerotic arteries from EC-Dicer^WT^ and EC-Dicer^flox^ mice fed a HFD for 12 weeks were performed (*n*=2 mice per group; P<0.05 by a moderated *t*-test (Limma); fold change cutoff=1.2). Overall, 469 transcripts were upregulated and 652 transcripts were downregulated in EC-Dicer^flox^ mice compared with EC-Dicer^WT^ mice. In addition to the transcription factor *c-Myb*, the Wnt pathway members *transcription factor 7 (Tcf7)* and *lymphoid enhancer-binding factor 1* (*Lef1)* and the expression of EC-specific genes, such as *Cadherin 5* (*Cdh5;* also known as VE-Cadherin), *Claudin-5 (Cldn5)*, *BMX non-receptor tyrosine kinase (Bmx)* and *SRY (sex-determining region Y)-box 17 (Sox17)*, were increased in EC-Dicer^flox^ mice (Fig. 6a). By contrast, the expression level of proinflammatory genes, like *Ccl2*, *CD44 antigen (CD44)*, *Rho-associated coiled-coil containing protein kinase 1* (*Rock1)* and *Nfkb1*, was reduced in the aortas of EC-Dicer^flox^ mice (Fig. 6a). We confirmed by qRT–PCR in a larger number of mice that endothelial Dicer deficiency increases the expression level of *c-Myb*, *Tcf7*, *dickkopf homolog 2* (*Dkk2)* and *Sox17*; however, the expression of *Lef-1* only tended to be increased in EC-Dicer^flox^ mice (Fig. 6b). Next, we analysed the differentially expressed genes using Ingenuity Pathway Analysis software. Genes related to biological processes such as development of blood vessels, inflammatory response, chemotaxis and immune cell adhesion were enriched among the differentially expressed genes in EC-Dicer^flox^ mice (Fig. 6c). Moreover, the expression levels of genes related to the KLF4 pathway were also differentially expressed in EC-Dicer^flox^ mice and were indicative of increased KLF4 signalling (Fig. 6d). KLF4 has been implicated in the regulation of *TCF7* and *MYB* expression^46^,^47^. Overexpression and silencing of *KLF4* in HAECs increased and reduced the expression of *c-MYB*, respectively, whereas the expression of *TCF7* mRNA was not affected (Supplementary Figs 7 and 8). Accordingly, knockout of *Dicer* in ECs resulted in *c-MYB* expression in ECs covering aortic root lesions, which was not detectable in ECs from EC-Dicer^WT^ mice by dual immunostaining of c-MYB and vWF (Supplementary Fig. 9). Overall, these data demonstrate that loss of Dicer upregulates the expression of KLF4-regulated genes in ECs, such as *c-MYB*.

Although deficiency of endothelial Dicer preferentially regulated KLF4-dependent genes, KLF4 mRNA expression levels in the aortic wall were not different between EC-Dicer^flox^ and EC-Dicer^WT^ mice (Fig. 6e). However, the number of KLF4-expressing arterial ECs was increased in EC-Dicer^flox^ compared with EC-Dicer^WT^ mice (Fig. 6f), compatible with translational inhibition of KLF4 expression by miR-103. These results suggest that activation of the KLF4 pathway in ECs contributes to the atheroprotective effect of endothelial Dicer deficiency by reducing arterial inflammation and increasing endothelial differentiation.

### miR-103 induces endothelial inflammation by targeting KLF4

**Figure 7:**
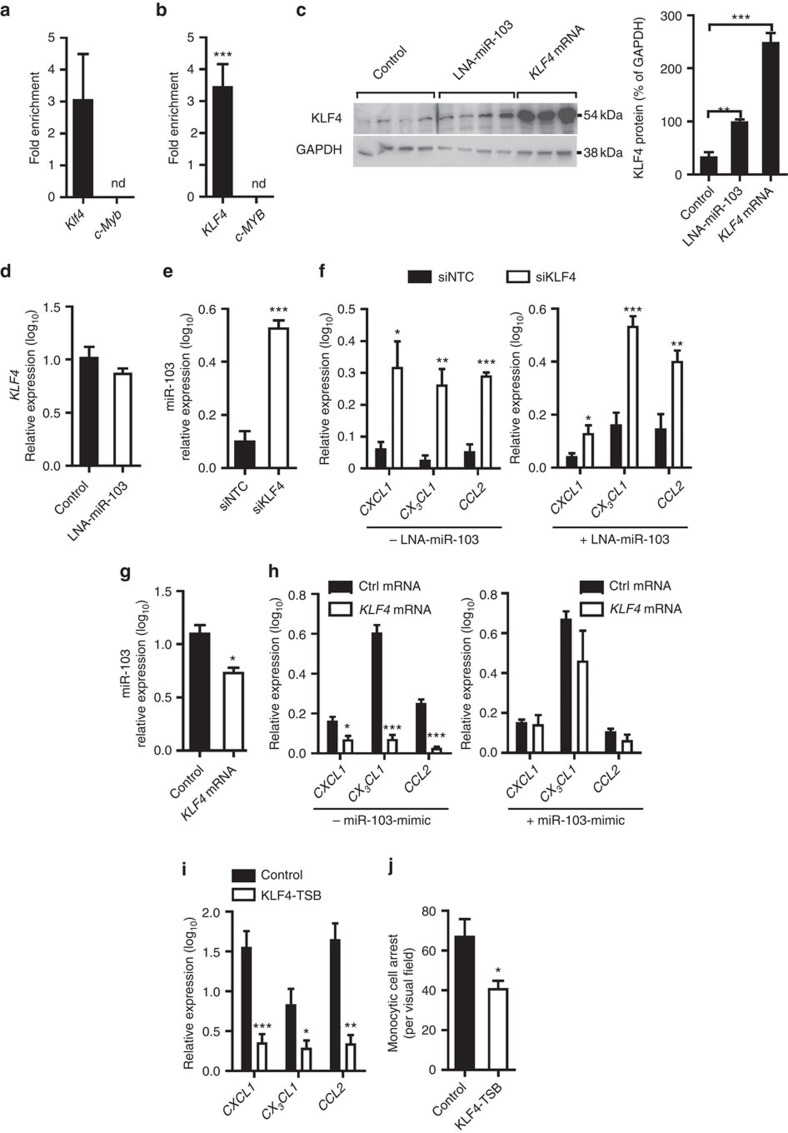
miR-103 promotes inflammatory activation of ECs by targeting KLF4. (**a**,**b**) Enrichment of *Klf4* and *c-Myb* transcripts in the miRNA-induced silencing complex (RISC) of mouse aortic ECs (**a**) and human aortic ECs (HAECs, **b**) treated with miR-103-mimics as determined by GW182-IP. The results are expressed as the fold enrichment of the mRNAs in GW182-IP samples compared with the input samples. Results of two independent experiments are shown. ND indicates not detected. (**c**) Immunoblot analyses of KLF4 protein expression in HAECs treated with LNA-inhibitors of miR-103, non-targeting control LNA-oligonucleotides or premade *KLF4* mRNA (*n*=3–4 per group). The KLF4 protein levels were normalized to those of GAPDH. Full scans of western blots are provided in Supplementary Fig. 14. (**d**) *KLF4* mRNA levels in HAECs treated with LNA-inhibitors of miR-103 or control LNA-oligonucleotides (*n*=4–5 per group). (**e**) MiR-103 expression after silencing KLF4 using siRNA (siKLF4) in HAECs. A non-targeting siRNA (siNTC) was used in the control group (*n*=4–5 per group). (**f**) The expression of *CXCL1*, *CX*_*3*_*CL1* and *CCL2* after silencing KLF4 (siKLF4) in HAECs treated with (right) or without (left) LNA-inhibitors of miR-103 (*n*=4–5 per group). siNTCs were used in the control group. (**g**,**h**) The effect of transfection with *GFP* mRNAs (Ctrl) or premade *KLF4* mRNAs on miR-103 (**g**) and chemokine expression (**h**) in HAECs treated with (**h**, right) or without (**g**; **h**, left) miR-103-mimics (*n*=3–5 per group). (**i**) Expression of *CXCL1*, *CX*_*3*_*CL1* and *CCL2* in HAECs treated with LNA-oligonucleotides (KLF4-target site blockers; KLF4-TSBs) designed to inhibit the interaction between miR-103 and the 3′UTR of KLF4 (*n*=6 per group). Non-targeting LNA-oligonucleotides were used in the control group. (**j**) Flow chamber assays to determine monocyte adhesion to HAECs treated with KLF4-TSBs or non-targeting oligonucleotides (*n*=4 per group). The data are represented as the mean±s.e.m. of the indicated number (*n*) of repeats. *P<0.05, ** *P*<0.01 and *** *P*<0.001 by Student's *t*-test (**a**,**b**,**d**–**j**) and one-way analysis of variance (**c**).

Notably, miR-103 can directly target the *KLF4* mRNA by binding to a conserved site in its 3′ untranslated region (UTR; Supplementary Fig. 10)^48^. Overexpressing a mutant GW182 protein in murine (Fig. 7a) and human ECs (Fig. 7b) enabled the locking of miRNAs and their targets in the RISC^49^. MiR-103-mimic treatment in these cells enriched *KLF4* mRNA but not *c-MYB* mRNA in the GW182-IPs, demonstrating that miR-103 targets *KLF4*, but not *c-MYB* mRNA in ECs (Fig. 7a,b). Moreover, treatment of HAECs with LNA inhibitors of miR-103 increased KLF4 protein expression (Fig. 7c) but did not affect *KLF4* mRNA levels (Fig. 7d). By contrast, the expression of KLF2 protein was not affected by treatment of HAECs with LNA inhibitors of miR-103 (Supplementary Fig. 11). Taken together, these results suggest that reduced endothelial miR-103 expression due to Dicer deficiency results in increased KLF4 protein expression in atherosclerotic endothelium.

Silencing of KLF4 in HAECs upregulated the expression level of miR-103 (Fig. 7e), as well as those of the *CXCL1*, *CX*_*3*_*CL1* and *CCL2* mRNAs in both the presence and absence of miR-103 inhibition (Fig. 7f). Conversely, overexpression of KLF4 downregulated the expression levels of miR-103 (Fig. 7g) and the *CXCL1*, *CX*_*3*_*CL1* and *CCL2* mRNAs (Fig. 7h) in HAECs, and treatment with miR-103 mimics prevented the suppression of chemokine expression by KLF4 (Fig. 7h). These data suggest that KLF4 affects chemokine expression downstream of miR-103, but may also act upstream of this miRNA in a negative feedback loop. TNF-α stimulation and blockage of NF-κB reduced and increased the expression of *KLF4*, respectively (Supplementary Fig. 12). Moreover, inhibition of the interaction between miR-103 and its binding site in the 3′UTR of *KLF4* using LNA-modified target site blockers (KLF4-TSBs) reduced the expression levels of chemokine mRNAs (Fig. 7i) and diminished monocyte adhesion (Fig. 7j), demonstrating that miR-103 regulates the adhesive properties of ECs by targeting KLF4. Overall, these results indicate that miR-103 induces chemokine expression in ECs by translational repression of the anti-inflammatory transcription factor KLF4.

### Targeting of KLF4 by miR-103 promotes atherosclerosis

**Figure 8:**
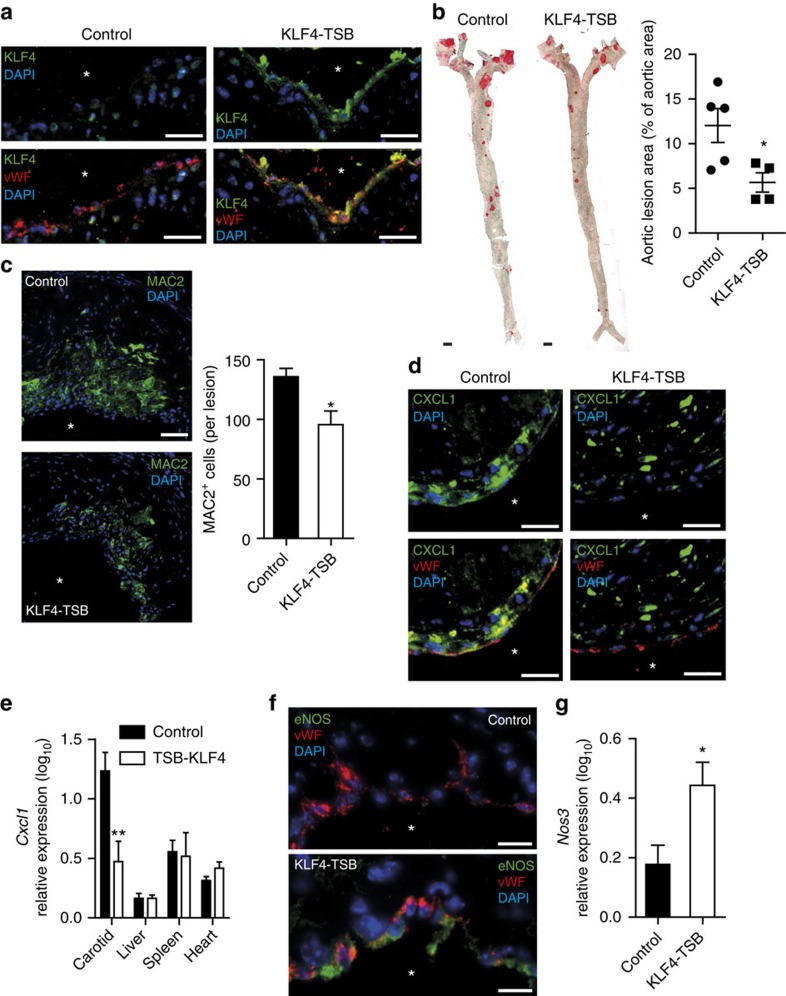
Inhibition of the interaction between miR-103 and KLF4 limits atherosclerosis. (**a**) Immunostaining of KLF4 and von Willebrand factor (vWF) in aortic root sections of *Apoe*^*−/−*^ mice treated with KLF4-TSBs or non-targeting LNA-oligonucleotides (control). Representative images are shown. Atherosclerosis (**b**) quantified in Oil red O-stained, *en face* prepared aortas and the lesional macrophage cell number (**c**) determined by MAC2 immunostaining in aortic root lesions from *Apoe*^*−/−*^ mice treated with KLF4-TSBs or control oligonucleotides (*n*=4–6 mice per group). (**d**) Dual immunostaining of CXCL1 and vWF in aortic root sections of *Apoe*^*−/−*^ mice treated with KLF4-TSBs or control oligonucleotides. (**e**) The expression levels of *Cxcl1* in the carotid, liver, spleen and heart of KLF4-TSB-treated *Apoe*^*−/−*^ mice compared with control mice (*n*=4–7 mice per group). (**f**) Dual immunostaining of eNOS and vWF in aortic root sections of *Apoe*^*−/−*^ mice treated with KLF4-TSBs or control oligonucleotides. (**g**) *Nos3* mRNA expression in the carotid arteries of KLF4-TSB-treated *Apoe*^*−/−*^ mice compared with control mice (*n*=5–6 mice per group). The nuclei were counterstained with 4',6-diamidino-2-phenylindole (DAPI). Asterisks indicate the lumen. Representative images of three independent experiments are shown. Scale bars, 10 μm (**f**), 25 μm (**a**,**d**), 50 μm (**c**) and 1 mm (**b**). The data are represented as the mean±s.e.m. of the indicated number (*n*) of repeats. *P<0.05 and **P<0.01 by Student's *t*-test.

To study the effect of the interaction between miR-103 and KLF4 on atherosclerosis, *Apoe*^*−/−*^ mice were treated with KLF4-TSBs or control LNA-modified olignonucleotides during the last 4 weeks of an 8-week HFD feeding programme. Increased endothelial expression of KLF4 in *Apoe*^*−/−*^ mice treated with KLF4-TSBs was detected by dual immunostaining of KLF4 and vWF (Fig. 8a). Treatment with KLF4-TSBs reduced the aortic lesion area (by 53%; Fig. 8b) and the lesional macrophage number (by 30%; Fig. 8c) compared with the treatment with control oligonucleotides. Moreover, inhibition of the interaction between miR-103 and KLF4 reduced the number of CXCL1-expressing ECs (Fig. 8d) and downregulated the *Cxcl1* mRNA expression in carotid arteries (Fig. 8e). By contrast, the expression level of *Cxcl1* was not affected in other tissues, like the liver, spleen and heart, following KLF4-TSBs treatment (Fig. 8e). Treatment with KLF4-TSBs induced endothelial expression of eNOS in aortic root lesions and the expression of *Nos3* in carotid arteries (Fig. 8f,g). Serum cholesterol levels were similar between control mice and mice treated with KLF4-TSBs (Supplementary Fig. 13). Taken together, these findings indicate that miR-103 enhances atherosclerotic lesion formation by suppressing KLF4.

## Discussion

Here, we demonstrate that deficiency of Dicer in the endothelium of *Apoe*^*−/−*^ mice diminishes endothelial inflammation, which reduces monocyte adhesion to atherosclerosis-prone endothelium and the development of atherosclerotic lesions. Deletion of *Dicer* in ECs primarily decreased the expression of a small subset of unstable miRNAs, including miR-103, and promoted KLF4-dependent gene expression. MiR-103 expression was upregulated by NF-κB and mildly oxidized LDL in human ECs and increased the expression of CXCL1 by targeting KLF4, thereby enhancing monocyte adhesion to ECs. Inhibition of the miR-103-KLF4 interaction reduced atherosclerosis, lesional macrophage accumulation and endothelial CXCL1 expression in the arteries of *Apoe*^*−/−*^ mice, indicating a proinflammatory and proatherogenic role of miR-103 by targeting KLF4.

The normal functions of various differentiated cell types, such as cardiomyocytes, pancreatic beta cells and SMCs, requires Dicer activity and fully functioning miRNA biogenesis^32^,^33^,^50^. However, mice with a deletion of the *Dicer* gene in ECs develop normally, indicating that permanent Dicer activity is of less importance in EC homeostasis^37^. Notably, knockdown of Dicer in ECs *in vitro* reduces the expression of CXCL1 and upregulates endothelial genes, like *eNOS* and *Tie2*, indicating that Dicer promotes inflammatory activation and impairs endothelial differentiation^38^,^51^. ECs at arterial bifurcations are primed by disturbed flow for inflammatory activation in response to hyperlipidemic stress, which results in NF-κB-dependent upregulation of CXCL1 and atherogenic monocyte adhesion^8^,^15^. Accordingly, we found that endothelial Dicer deficiency downregulates CXCL1 in murine arteries and reduces the adhesion of monocytes to the endothelium during the early stage of atherosclerosis. In line with its effect on monocyte adhesion, deficiency of Dicer in ECs reduced the development of atherosclerosis and the accumulation of lesional macrophages, suggesting that the generation of miRNAs in ECs during atherosclerosis promotes lesion formation by increasing CXCL1-dependent monocyte adhesion. The detrimental role of endothelial Dicer during atherogenesis may be due to the key role of miRNAs in the maladaptive response of ECs to disturbed flow. The miRNA expression profile in ECs exposed to disturbed blood flow at arterial bifurcations differs substantially from that in ECs at unbranched arterial segments^42^ and is characterized by downregulation of atheroprotective (such as miR-126-5p)^52^ and upregulation of pro-atherogenic miRNAs (such as miR-92a)^41^. Moreover, ECs at arterial bifurcations proliferate more frequently than quiescent ECs at unbranched segments^52^. In contrast to proliferating cells, the majority of miRNAs in quiescent cells is stable (persistence of miRNAs up to 2 months following Dicer ablation in neurons *in vivo* have been reported)^53^, stored in Argonaut complexes and functionally silent probably due to their sequestration by polysomes^29^,^54^,^55^,^56^. Therefore, reduced endothelial miRNA biogenesis at predilection sites of atherosclerosis may cause the protective effects of endothelial Dicer deficiency on lesion formation.

Although miRNAs are generally stable, a small subset of miRNAs that associates with Argonaut proteins demonstrates fast turnover rates in a highly dynamic and cell type-specific manner^28^,^29^,^57^. After silencing of Dicer in ECs, let-7 family miRNAs, miR-103, miR-221 and miR-27b have a higher turnover rate than miR-126-3p, miR-21, miR-23a and miR-26a^39^. We found that the expression levels of four miRNAs, including miR-103, were highly dependent on endothelial Dicer activity. Hence, downregulation of unstable miRNAs, like miR-103, following *Dicer* deletion may limit monocyte adhesion and atherosclerosis in mice. MiR-103 has a key role in regulating insulin sensitivity in adipocytes by targeting caveolin-1, and in cancer cell growth and metastasis by repressing different targets, including KLF4 (refs 48, 58, 59). MiR-103 is one of the most highly expressed miRNA in ECs cultured under static conditions and downregulated by high-shear stress^38^,^60^. *In vivo*, miR-103 is upregulated at predilection sites for the development of atherosclerosis characterized by disturbed flow-induced endothelial NF-κB activation^42^. The results presented here show that NF-κB activation upregulates miR-103 and thereby promotes the expression of chemokines, such as CXCL1, CCL2 and CX_3_CL1. MiR-103 increased CXCL1-dependent monocyte adhesion and rescued the decreased chemokine expression caused by knockdown of *Dicer* in ECs, indicating that reduced endothelial miR-103 levels limit monocyte adhesion and atherosclerosis in EC-Dicer^flox^ mice by downregulating CXCL1.

The reduced endothelial inflammation in atherosclerotic arteries of EC-Dicer^flox^ mice was associated with enhanced KLF4 activity. Although KLF4 is closely related to KLF2, only 30% of the KLF4-regulated genes in ECs are also controlled by KLF2, indicating non-redundant roles of KLF2 and KLF4 in endothelial function^46^. For example, KLF4 but not KLF2 upregulates Cdh5 and Claudin-5 by direct interaction with their promoter and thus reduces endothelial permeability^61^,^62^,^63^. By contrast, both KLF4 and KLF2 attenuate endothelial inflammation by inhibiting NF-κB activation through competitive binding to the p300 coactivator^2^. An atheroprotective role of endothelial KLF4 was demonstrated by gain-an-loss-function studies in mice^64^. The expression of KLF2 and KLF4 is transcriptionally upregulated by the MEK5/Erk5/MEF2 signalling pathway and posttranscriptionally silenced by disturbed flow-induced miR-92a^3^,^65^. In accordance with previous reports in cancer cells, we found that miR-103 represses the translation of KLF4 mRNA by direct interaction with a conserved binding site in its 3′UTR, which results in NF-κB-mediated upregulation of CXCL1 in ECs^48^. Hence, miR-103 might act as a molecular switch for the inflammatory activation of arterial ECs by fine-tuning the functional antagonism between KLF4 and NF-κB. The findings that inhibition of the interaction between miR-103 and KLF4 reduced atherosclerosis and increased endothelial KLF4 expression in *Apoe*^*−/−*^ mice demonstrates a proatherogenic role of this interaction and suggests that derepression of KLF4 due to downregulation of miR-103 contributes to the atheroprotective effect of Dicer deficiency in ECs.

In conclusion, deficiency of Dicer in the endothelium of *Apoe*^*−/−*^ mice reduced monocyte adhesion to the early atherosclerotic endothelium by downregulating CXCL1, and thereby diminished lesion formation. Thus, endothelial Dicer activity at arterial sites predisposed to atherosclerosis may play a pro-atherogenic role by generating proinflammatory miRNAs. This effect of Dicer is attributable to reduced endothelial miR-103 expression and the subsequent restoration of *KLF4* expression. Moreover, selective inhibition of the targeting of KLF4 by miR-103 using antisense oligonucleotides may represent a novel approach to treat atherosclerosis.

## Methods

### Animal models

Cdh5-CreER^T2^ mice (kindly provided by Dr Iruela-Arispe, UCLA, Los Angeles, CA, USA) were crossed with Dicer1^flox/flox^/*Apoe*^*−/−*^ mice (Jackson Laboratory) to obtain Cdh5-CreER^T2^Dicer1^WT/flox^/*Apoe*^*−/−*^ mice^66^,^67^. Cdh5-CreER^T2^Dicer1^flox/flox^/*Apoe*^*−/−*^ (EC-Dicer^flox^) and Cdh5-CreER^T2^Dicer1^WT/WT^/*Apoe*^*−/−*^ (EC-Dicer^WT^) littermates were used for experiments. Cre recombinase activity was induced by intraperitoneal injection of the mice with TMX (2 mg per 20 g body weight; Sigma-Aldrich) dissolved in neutral oil (Migyol; Sasol) for 5 consecutive days. Deletion of the conditional *Dicer* allele after TMX injection was verified in the aortas of EC-Dicer^flox^ mice by PCR^67^. One week after the last TMX injection, 6- to 8-week-old female mice were fed a HFD consisting of 21% crude fat, 0.15% cholesterol and 19.5% casein (Altromin, Lage, Germany) for the indicated time points. For immunohistochemistry, the aortas and carotid arteries were harvested after *in situ* perfusion fixation with 4% paraformaldehyde (Carl Roth) or PAXgene (Qiagen). For purification of mRNAs or miRNAs, the arteries were perfused with RNAlater (Life Technologies). All animal experiments were reviewed and approved by the local authorities (State Agency for Nature, Environment and Consumer Protection of North Rhein-Westphalia and District Government of Upper Bavaria) in accordance with the German animal protection laws.

### Histology and immunostaining

Thoracoabdominal aortas were prepared *en face* and stained with Oil Red O stain. The Oil Red O-positive area was quantified from digital images of the aorta using image analysis software (ImageJ). Serial sections (5 μm thick) from carotid arteries and aortic roots were stained with Movat's pentachrome or Elastic van Gieson stain. A bright-field microscope (Leica DM6000B; Leica Microsystems) connected to a CCD camera (Leica DFC365FX) was used to obtain the images. The lesion area was quantified using planimetry (Leica LAS software). Immunostaining of CXCL1 (1:25; rabbit polyclonal antibody, PeproTech), macrophage-specific Mac2 (MAC2; 1:400; clone M3/38, Cedarlane), α-smooth muscle actin (1:200; clone 1A4, Dako), CD31 (1:75; goat polyclonal antibody, Santa Cruz Biotechnology), vWF (1:1,000; rabbit polyclonal antibody, Abcam), KLF4 (1:200; rabbit polyclonal antibody, Abcam), eNOS (1:100; purified mouse antibody, BD Bioscience) and c-MYB (1:300, rabbit polyclonal antibody, Hölzel Diagnostika Handels GmbH) was performed in carotid arteries or aortic root sections. The staining of KLF4 and CD31 was performed separately on adjacent sections. Cell nuclei were counterstained with 4′,6-diamidino-2-phenylindole (Vectashield, Vector Laboratories). The positively stained area or the number of positive cells was normalized to the lesion area or lesional cell number, respectively, using ImageJ. Non-specific primary antibodies were used as negative controls (Santa Cruz Biotechnology). The background of the negative control defined the threshold. The analysis of the stainings was performed in blinded manner.

### Human carotid lesion samples

Human atherosclerotic lesion samples were obtained during carotid endarterectomy and fixed with 4% paraformaldehyde (Carl Roth). The Ethics Committee of the Medical Faculty at RWTH Aachen University approved the study protocol for the collection of human atherosclerotic plaque specimens and all participants gave their written informed consent.

### Blood chemistry

Serum cholesterol levels were measured by dry chemistry using a Vitros 250 Analyzer (Ortho Clinical 10 Diagnostics).

### *Ex vivo* perfusion of carotid arteries

The left carotid arteries were isolated from EC-Dicer^flox^ and EC-Dicer^WT^ mice after 4 weeks of HFD feeding and were mounted onto a microscopic stage. Monocytic Mono Mac6 cells (MM6; 10^6^ cells per ml; Leibniz Institute DSMZ-German Collection of Microorganisms and Cell Cultures, Braunschweig, Germany) labelled with calcein AM (1 μM; Life Technologies), were perfused at a flow rate of 4 μl min^−1^ and monocytic cell adhesion was recorded using stroboscopic epifluorescence illumination^15^.

### *In vivo* target site blocker treatment

Following 4 weeks of HFD feeding, *Apoe*^*−/−*^ mice were randomized to the different experimental groups and injected once weekly via the tail vein with KLF4 LNA-target site blocker oligonucleotides (5′- GTATGCAGCAGTTGG -3′) or control LNA-target site blocker oligonucleotides (5′- GCTCCCTTCAATCCAA -3′; 0.4 mg per 20 kg; miRCURY LNA Target Site Blocker, *in vivo* use; Exiqon). During the injection period, mice were fed with a HFD. The tissues were harvested 1 week after the last injection.

### MiRNA real-time PCR array

After reverse transcription and pre-amplification of total RNA using the Megaplex RT & Preamp Rodent Pool Set (Life Technologies), the samples were loaded onto preconfigured 384-well microfluidic TaqMan Array MicroRNA Cards for real-time PCR (RT–PCR) analyses of 641 mouse miRNAs, using the 7900HT Real-Time PCR System (all from Life Technologies). Data analysis was performed using StatMiner software (Integromics) along with multiple internal control genes and the cycle threshold (CT) method. After 4 and 12 weeks HFD feeding, a total of 428 and 458 miRNAs, respectively, were detected according to the detection limit (defined as a CT=40) of the individual assays.

### Global gene expression analysis

Global gene expression analysis was performed using Agilent 8 × 60 K SurePrint G3 Mouse Gene Expression in combination with a one-colour-based hybridization protocol (IMGM Laboratories GmbH). Fluorescent signals on the microarrays were detected using the Agilent DNA Microarray Scanner (Agilent Technologies Germany GmbH). Flagged genes were removed from the analysis. Probe set intensities were summarized and normalized using robust multi-array average, and significant differential expression was determined by moderated *t*-test (Limma) using a *P*-value cutoff of 0.05 and a fold change cutoff of 1.2 (GeneSpring GX Software, Agilent). Differentially regulated genes were analysed by Ingenuity Pathway Analysis (Qiagen). The Fisher's exact *t*-test was selected for the function and pathway analysis.

### Preparation of moxLDL

Human native low-density lipoprotein (nLDL; 1 mg ml^−1^, Calbiochem, Millipore) was incubated with 5 μM CuSO_4_ at 37 °C for 4 h to prepare mildly oxidized-low-density lipoprotein (moxLDL). The LDL oxidation was stopped by adding 10 μM EDTA and the LDL was passed through PD-10 desalting column (GE Healthcare). To prepare nLDL for experimental use as a negative control, all the above steps were performed except the addition of CuSO_4_. The moxLDL and nLDL were used within 14 days after preparation and stored at 4 °C.

### Cell culture

HAEC (Cat. # C-12271; PromoCell), umbilical vein ECs (HUVEC; Cat. # CC-2517; Lonza) and mouse aortic ECs (Pelobiotech) were cultured in collagen-coated dishes (Millipore) using EC-growth medium (PromoCell). HAECs were stimulated for 6 h with TNF-α (10 ng ml^−1^; R&D Systems), nLDL (50 μg ml^−1^; Calbiochem, Millipore) or moxLDL (50 μg ml^−1^, Calbiochem, Millipore). HAECs were incubated for 30 min with 5 μM BAY11-7085 (Millipore) to block NF-κB activation. Subsequently, the medium was replaced and RNA was isolated after 24 h. HAECs were cultured in collagen-coated perfusion chambers (μ-Slides VI^0.4^, ibidi GmbH) and exposed to high-shear stress (15 dyne cm^−2^) or low-shear stress (4 dyne cm^−2^) for 48 h generated by perfusion with medium (ibidi Pump System, ibidi GmbH).

Gram-negative endotoxin levels were tested in the medium of HAECs und HUVECs using the LAL Chromogenic Endotoxin Quantitation Kit (Thermo Scientific). The endotoxin concentration was consistently less than 0.1 Endotoxin Units (EU)/ml.

MM6 cells (Mono Mac 6; Leibniz Institute DSMZ-German Collection of Microorganisms and Cell Cultures) were cultured in RPMI medium (with L-Glutamine; GE Healthcare Life Sciences) containing 10% FBS, OPI media supplement (1 mM oxaloacetate, 0.45 mM pyruvate, 0.2 U ml^−1^ insulin; Sigma-Aldrich), 1 × MEM non-essential amino acids (Life Technologies), 2.5 μg ml^−1^ Plasmocin (InvivoGen), 100 μg ml^−1^ streptomycin and 100 U ml^−1^ penicillin (Life Technologies).

To isolate ECs, the periadventitial fat and connective tissue was removed from the aortas of EC-Dicer^WT^ and EC-Dicer^flox^ mice 2 weeks after TMX injection. The tissue was cut into 1–2 mm^2^ sections, which were cultured in endothelial growth medium (PromoCell) for 7 days to allow the outgrowth of ECs. Subconfluent ECs of passage 3–5 were used for experiments. The EC phenotype was confirmed by lectin staining (*Lycopersicon esculentum*; Sigma)^15^.

Myeloid cells were isolated from the blood of EC-Dicer^WT^ and EC-Dicer^flox^ mice 4 weeks after TMX injection and HFD feeding by magnetic cell sorting using CD11b^+^ microbeads (MACS Miltenyi Biotec GmbH).

### Treatment of HAECs

Lipofectamine RNAiMAX (Life Technologies) was used to transfect HAECs with a LNA-miR-103 inhibitor (50 nM, miRCURY LNA microRNA Inhibitors; Exiqon), a miR-103 mimic (15 nM, *mir*Vana mimics; Life Technologies), Dicer GapmeRs (10 nM, LNA GapmeRs; Exiqon), miR-103-KLF4 target site blockers (50 nM miRCURY LNA microRNA Target Site Blockers; Exiqon), premade *KLF4* mRNA (2 μg, mRNAExpress Human KLF4 Transcript; BioCat GmbH) or scrambled controls. Total RNA was isolated after 24 or 48 h using the RNeasy Mini Kit (Qiagen) or mirVana Isolation Kit (Life Technologies). HAECs were transfected with a small interfering RNA (siRNA) against KLF4 or a non-targeting siRNA (1 μM Accell siRNA in Accell Delivery Cell Culture Medium; Thermo Scientific) for 72 h. Additional treatment with the LNA-miR-103 inhibitor was performed as described above.

### Quantitative real-time PCR

Total RNA was isolated from carotid arteries, aortas or cultured ECs using the mirVana miRNA Isolation Kit (Life Technologies), the RNeasy Mini Kit (Qiagen) or NucleoSpin microRNA Kit (Macherey-Nagel GmbH & Co. KG) and reverse-transcribed with a TaqMan microRNA reverse transcription kit or a high-capacity cDNA reverse transcription kit (both from Life Technologies). MiRNA qRT-PCR was performed using TaqMan microRNA assays and TaqMan Universal PCR Master Mix (both from Life Technologies). qRT–PCR assays for mRNAs were performed either with TaqMan gene expression assays and TaqMan Universal PCR Master Mix (both from Life Technologies) or with gene-specific primers (Sigma-Aldrich; Supplementary Table 3) and a SYBR Green Master Mix (Thermo Scientific). All qRT–PCR experiments were run on a 7900HT real-time PCR system (Life Technologies). The data were normalized to single or multiple reference genes (sno-135, RNU44 and U6 for miRNAs; *GAPDH* and *B2M* for mRNAs) and scaled to the sample with of the lowest expression using Qbase^PLUS^ software (Biogazelle NV).

### *In situ* reverse transcriptase PCR

Sections (5 μm thick) of carotid arteries and aortic roots were treated with DNase (Roche) for 15–17 h at 37 °C. One-step reverse transcriptase *in situ* PCR was performed using gene-specific Taq *in situ* primers (Sigma-Aldrich; Supplementary Table 3), SuperScript One-Step RT–PCR with PlatinumTaq (Life Technologies), and digoxigenin-11-dUTPs (Roche)^68^. After two washes with saline-sodium citrate buffer buffer and blocking of biotin/avidin-binding sites (Blocking Kit; Vector Laboratories), the sections were incubated with horseradish peroxidase-conjugated anti-digoxigenin sheep F'ab fragments (Roche) for 1 h at 37 °C. The probes were visualized using a tyramide-based amplification system (TSA Plus Biotin; Perkin Elmer) and DyLight 549-labelled streptavidin (Kirkegaard & Perry Laboratories)^52^.

### Argonaute 2 immunoprecipitation

ECs were harvested and washed in ice-cold phosphate-buffered saline (GE Healthcare Life Sciences), and then incubated in lysis buffer containing 400 μM vanadyl ribonucleoside complexes (New England Biolabs) and protease inhibitors (Complete Protease Inhibitor Cocktail Tablets; Roche).^26^ The cell lysates were centrifuged and the input RNA was extracted from the supernatant using TRIzol reagent (Life Technologies). A human monoclonal AGO2 Ab (5 μg per 1,000 μl; clone 2E12-1C9; Abnova) or control IgG (5 μg per 1,000 μl; Millipore) was pre-incubated with protein A/G-conjugated magnetic beads (Millipore), and then incubated with the cell extract for 7 h at 4 °C. The precipitate was immobilized with a magnetic separator (Millipore). RNA was isolated from the precipitate using TRIzol reagent, reverse-transcribed using a high-capacity cDNA reverse transcription kit (Life Technologies), and amplified with gene-specific primers (Supplementary Table 3) and SYBR Green PCR Master Mix (Thermo Scientific). The fold enrichment of target genes in AGO2-immunoprecipitates (AGO2-IPs) over IgG-immunoprecipitates (IgG-IPs) was calculated as follows: ΔCT_AGO2-IP_=CT_input_−CT_AGO2-IP_, ΔCT_IgG-IP_=CT_input_−CT_IgG-IP_, ΔΔCT=ΔCT_AGO2-IP_−ΔCT_IgG-IP_; and fold enrichment=2^ΔΔCT^.

### MiRNA target identification and quantification system (MirTrap)

HAECs and mouse aortic ECs were co-transfected with miR-103-mimics and pMirTrap Vector using the Xfect MicroRNA Transfection Reagent in combination with Xfect Polymer for 24 h (all from Clontech). The pMirTrap Vector expressed a DYKDDDDK-tagged GW182 protein, which enabled locking of the miRNA/mRNA complex into the RISC^49^. After 24 h, ECs were harvested and washed in ice-cold × 1 phosphate-buffered saline (GE Healthcare Life Sciences), and then incubated in lysis buffer (MirTrap System, Clontech) supplemented with protease inhibitors (Complete Protease Inhibitor Cocktail Tablets; Roche). The cell lysates were centrifuged and part of the input RNA was extracted from the supernatant using the NucleoSpin RNA XS Kit (Macherey-Nagel GmbH & Co. KG). Anti-DYKDDDDK-conjugated magnetic beads were washed twice with lysis/wash buffer containing 1 mM dithiothreitol, 0.1 U μl^−1^ RNase inhibitor and protease inhibitors (Complete Protease Inhibitor Cocktail Tablets; Roche) and blocked for 3 h at 4 °C with 1.25 mg ml^−1^ tRNA solution and 1.25 mg ml^−1^ BSA. To immunoprecipitate the DYKDDDDK-tagged RISC complex, blocked anti-DYKDDDDK beads were incubated with the cell extract for 2 h at 4 °C. Immobilization of the precipitates and subsequent RNA isolation was performed using the NucleoSpin RNA XS Kit (Macherey-Nagel GmbH & Co. KG). Reverse transcription of input and IP samples were performed using a high-capacity cDNA reverse transcription kit (Life Technologies), followed by the amplification with gene-specific primers (Supplementary Table 3) using the SYBR Green PCR Master Mix (Thermo Scientific). The fold enrichment was calculated according to the manufacturer's protocol. Efficiency of transfection has been determined by performing a control transfection using miR-132-mimic, the pMirTrap Vector and the pMirTrap Control Vector, which express an AcGFP1 fluorescein protein containing a miR-132 target sequence. The efficient enrichment of AcGFP1 fluorescein protein in the RISC was confirmed and compared to that of a non-miR-132 target gene, such as *Lef1* mRNA transcript.

### Flow adhesion assay

Mono Mac6 cells (MM6; 0.5 × 10^6^ cells per ml) were labelled with calcein AM (1 μM) and, for some experiments, were treated with an antibody against C–X–C chemokine receptor type 2 (20 μg ml^−1^, clone 48311) or an isotype control IgG (20 μg ml^−1^, clone 20102; both from R&D Systems). HAECs were cultured in collagen-coated cell culture dishes (35 × 10 mm^2^; Becton Dickinson) and transfected with LNA-miR-103, miR-103 mimic, KLF4 target site blockers or scrambled controls for 24 h. MM6 cells were resuspended in medium containing 1 × Hank's Balanced Salt Solution (Life Technologies), 1 M HEPES (Thermo Scientific) and 0.5% bovine serum albumin, and then perfused in a parallel plate flow chamber over a confluent monolayer of HAECs at a flow rate of 0.1 ml min^−1^. Monocytic cell arrest on the endothelial monolayer was visualized by videomicroscopy at × 10 magnifications. After a 2-min observation period, cells adhering to the HAEC monolayer were counted in at least ten different microscopic view fields within 8 min.

### Enzyme-linked immunosorbent assay

The CXCL1 protein concentration was determined in HAEC cell lysates with the GRO/MGS ELISA Development Kit (PeproTech). The absorbances were measured at 450 and 405 nm, respectively, on a microplate reader (Tecan).

### Western blot analysis

HAECs were lysed in RIPA buffer (Sigma-Aldrich) including protease inhibitors (Complete Protease Inhibitor Cocktail, Roche). Cell lysates were resolved on SDS–PAGE gels and then transferred to nitrocellulose membranes. Proteins were detected using primary antibodies against KLF4 (1:1,000, EPR3550(2)ABC, Abcam), KLF2 (1:75, ab139699, Abcam) and GAPDH (1:1,000, clone 6C5, Millipore), and horseradish peroxidase (HRP)-conjugated secondary antibodies (1:1,000, Goat Anti-Mouse IgG HRP Affinity Purified PAb, R&D Systems; Goat Anti-Rabbit IgG HRP Affinity Purified PAb, Santa Cruz). Protein bands were visualized using an enhanced chemiluminescence detection system (ECL Advance, GE Healthcare Life Sciences) and an LAS 3000 Imager (Fuji Photo Film Co., Ltd.) and were quantified using Multigauge software (Fuji Photo Film). Intensities of the KLF4 and KLF2 bands were expressed as a percentage of those of the GAPDH bands.

### Statistical analysis

The miRNA real-time PCR array data were analysed using StatMiner 4.2 software (Integromics) and are presented as mean values. All other data represent the mean±s.e.m. Student's *t*-tests and one-way analysis of variance followed by the Newman–Keuls *post-hoc* test were used for statistical comparisons between groups using Prism 6 software (GraphPad Software Inc.). Sample size was estimated using StatMate software (GraphPad Software Inc.). *P*<0.05 was considered statistically significant.

## Additional information

**Accession codes:** The microarray data have been deposited in the National Center for Biotechnology Information Gene Expression Omnibus database (http://www.ncbi.nlm.nih.gov/geo/) under accession numbers GSE53433 and GSE53435.

**How to cite this article:** Hartmann, P. *et al*. Endothelial Dicer promotes atherosclerosis and vascular inflammation by miRNA-103-mediated suppression of KLF4. *Nat. Commun.* 7:10521 doi: 10.1038/ncomms10521 (2016).

## Supplementary Material

Supplementary Information Supplementary Figures 1-15, Supplementary Tables 1-3 and Supplementary Reference
