# Corrigendum: Endothelial Dicer promotes atherosclerosis and vascular inflammation by miRNA-103-mediated suppression of KLF4

**DOI:** 10.1038/ncomms11907

**Published:** 2016-06-07

**Authors:** Petra Hartmann, Zhe Zhou, Lucia Natarelli, Yuanyuan Wei, Maliheh Nazari-Jahantigh, Mengyu Zhu, Jochen Grommes, Sabine Steffens, Christian Weber, Andreas Schober

Nature Communications 7: Article number: 10521 10.1038/ncomms10521 (2016); Published: 0232016; Updated: 06072016

This Article contains an error in the dose of LNA-target site blocker oligonucleotides that was administered. In the Methods section, under the subheading ‘*In vivo* target site blocker treatment' the dose ‘0.4 mg per 20 kg' should read ‘0.4 mg per 20 g'.

