# Supplementary material for: Endothelial Dicer promotes atherosclerosis and vascular inflammation by miRNA-103-mediated suppression of KLF4
Source: Nat Commun. 2016 Feb 3;7:10521. doi: 10.1038/ncomms10521 (PMC4742841; doi:10.1038/ncomms10521)
Supplement: Supplementary Information — Supplementary Figures 1-15, Supplementary Tables 1-3 and Supplementary Reference [file ncomms10521-s1.pdf]

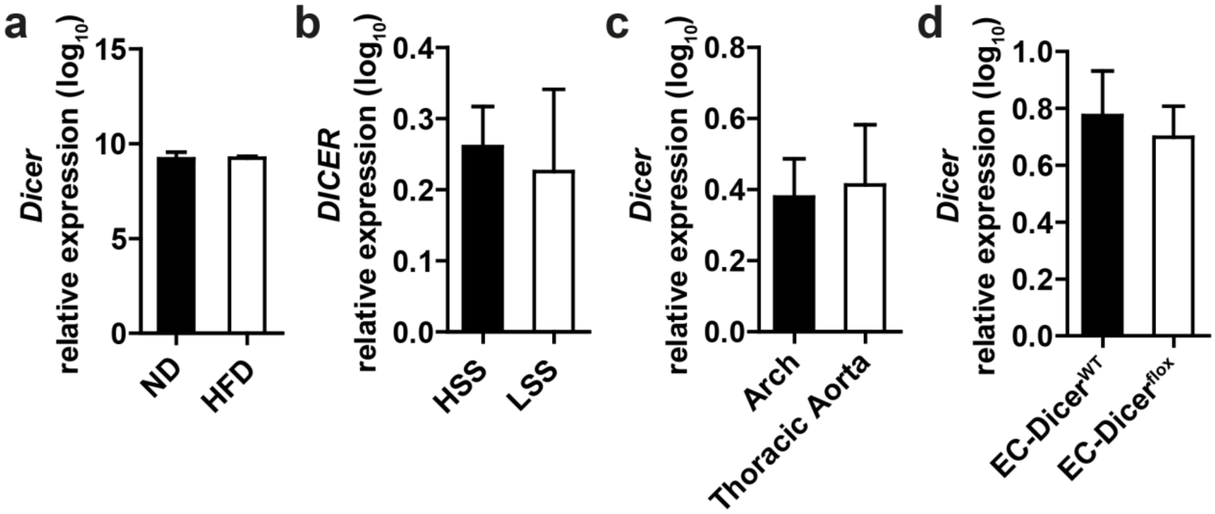

Supplementary Figure 1. Regulation of *Dicer* expression. (a) Quantitative RT-PCR analyses of *Dicer* mRNA expression in aortas of *Apoe*$^{-/-}$ mice fed a normal diet (ND) or a high fat diet (HFD) for 12 weeks (n = 3–4 mice per group). (b) Quantitative RT-PCR analyses of *Dicer* mRNA expression in human aortic ECs (HAECs) exposed to high shear stress (HSS) compared to low shear stress (LSS) for 48 h (n = 3–4 per group). (c) The expression levels of *Dicer* in the aortic arch and thoracic aorta of *Apoe*$^{-/-}$ mice fed a HFD (n = 4 mice per group). (d) *Dicer* mRNA expression in CD11b^{+} peripheral blood cells isolated from EC-Dicer^{WT} and EC-Dicer^{flox} mice fed a HFD for 4 weeks (n = 6 mice per group). The data are represented as the mean ± s.e.m. of the indicated number (n) of repeats. Student *t*-test was used to compare the groups.

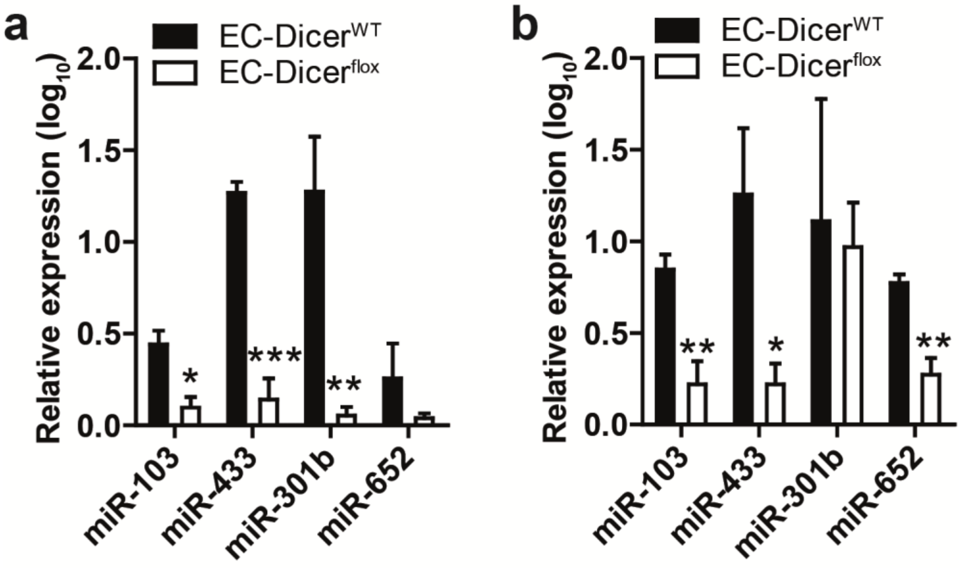

Supplementary Figure 2. Effects of endothelial Dicer deficiency on miRNA expression in different parts of the aorta. (a, b) The expression levels of miR-103, miR-301b, miR-433, and miR-652 in the aortic arch (a) and descending thoracic aorta (b) from EC-Dicer^{WT} compared to EC-Dicer^{flox} mice after HFD feeding (n = 3–4 mice per group). The data are represented as the mean ± s.e.m. of the indicated number (n) of repeats. *$P < 0.05$, **$P < 0.01$, and ***$P < 0.001$. Student *t*-test was used to compare the groups.

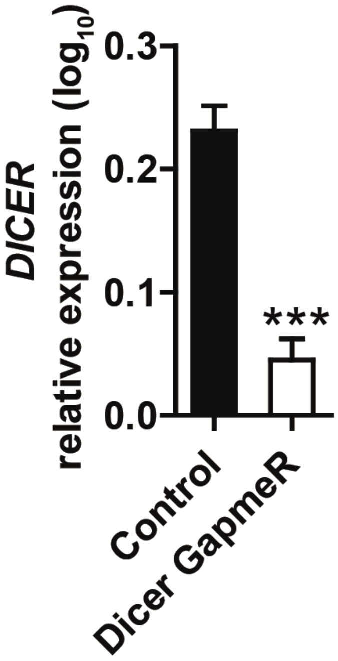

Supplementary Figure 3. Silencing of Dicer in ECs. Quantitative RT-PCR analyses of *DICER* mRNA expression in human aortic ECs (HAECs) treated with Dicer-specific LNA-GapmeRs or non-targeting control LNA-GapmeRs (n = 5 per group). The data are represented as the mean ± s.e.m. of the indicated number (n) of repeats. ***$P < 0.001$. Student *t*-test was used to compare the groups.

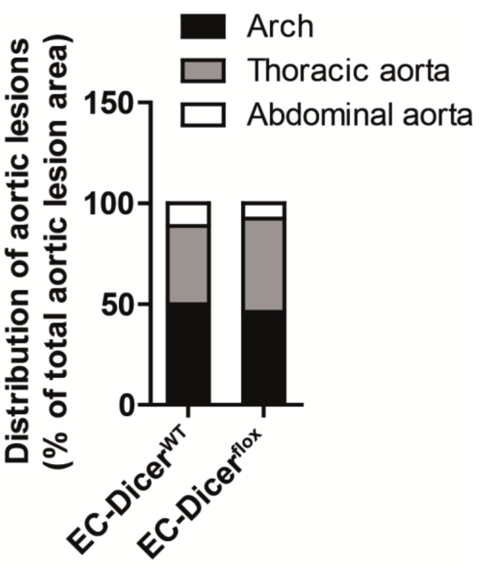

Supplementary Figure 4. Effects of endothelial Dicer deletion on the distribution of aortic lesions. Distribution of atherosclerotic lesions between the arch, thoracic aorta and abdominal aorta of EC-Dicer^{WT} and EC-Dicer^{flox} mice fed a HFD for 12 weeks (n = 5 mice per group). P = not significant. Student *t*-test was used to compare the groups.

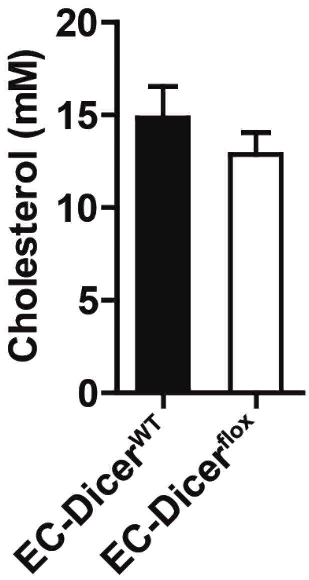

Supplementary Figure 5. Effect of endothelial Dicer knockout on serum cholesterol levels. Cholesterol levels were analyzed in the serum of EC-Dicer^{WT} and EC-Dicer^{flox} mice after 12 weeks of HFD feeding (n = 4–5 mice per group). The data are represented as the mean ± s.e.m. of the indicated number (n) of repeats. Student *t*-test was used to compare the groups.

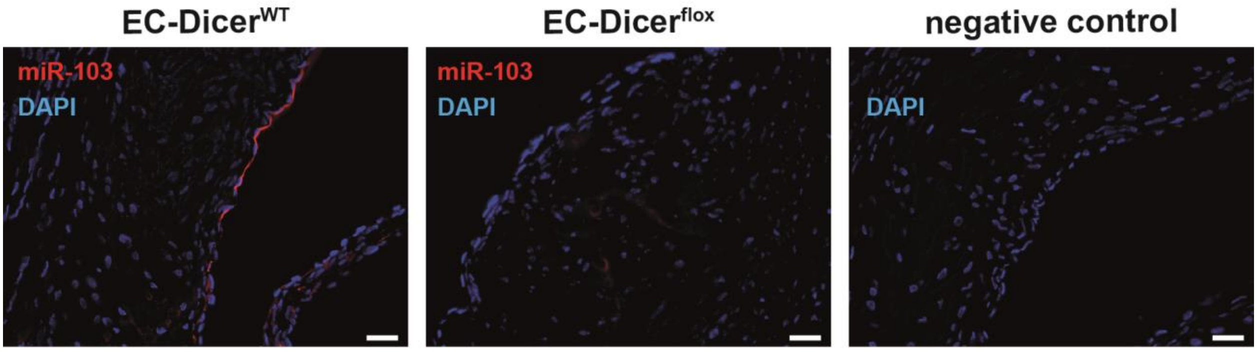

Supplementary Figure 6. MiR-103 expression in aortic root lesions. MiR-103 expression in aortic root lesions after 12 weeks of HFD feeding, as determined by *in situ* PCR. The nuclei were counterstained with DAPI. Negative control staining was performed without reverse transcription. Scale bar, 25 µm.

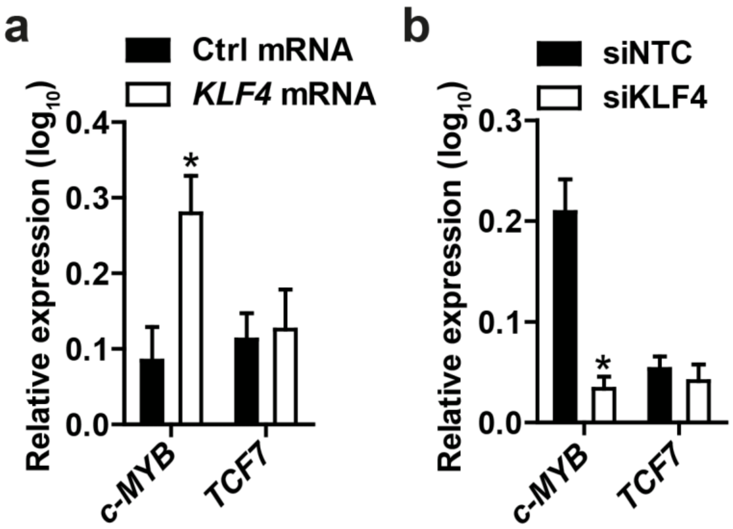

Supplementary Figure 7. Effects of KLF4 on the expression of *c-MYB* and *TCF7* in ECs. (a) Expression of *c-MYB* and *TCF7* mRNAs in HAECs treated with *GFP* control mRNA (Ctrl mRNA) or premade KLF4 mRNAs (n = 3–4 per group). (b) Expression of *c-MYB* and *TCF7* mRNAs in HAECs treated with siRNA against KLF4 (n = 4–5 per group). Non-targeting oligonucleotides were used as control. The data are represented as the mean ± s.e.m. of the indicated number (n) of repeats. *$P < 0.05$. Student *t*-test was used to compare the groups.

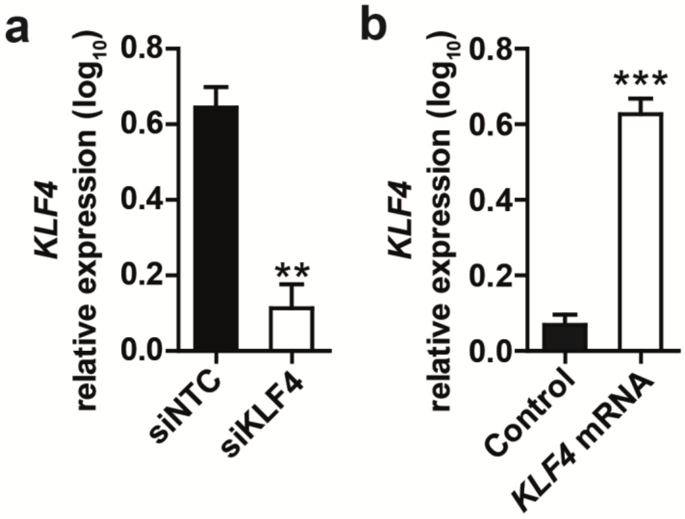

Supplementary Figure 8. Silencing and overexpression of KLF4 in ECs. (a) Quantitative RT-PCR analyses of *KLF4* mRNA expression levels in HAECs after silencing KLF4 (siKLF4). A non-targeting siRNA (siNTC) was used in the control group (n = 3–4 per group). (b) The effect of transfection with *GFP* mRNA (control) or premade *KLF4* mRNA on *KLF4* mRNA expression levels in HAECs (n = 3–5 per group). The data are represented as the mean ± s.e.m. of the indicated number (n) of repeats. **$P < 0.01$, and ***$P < 0.001$. Student *t*-test was used to compare the groups.

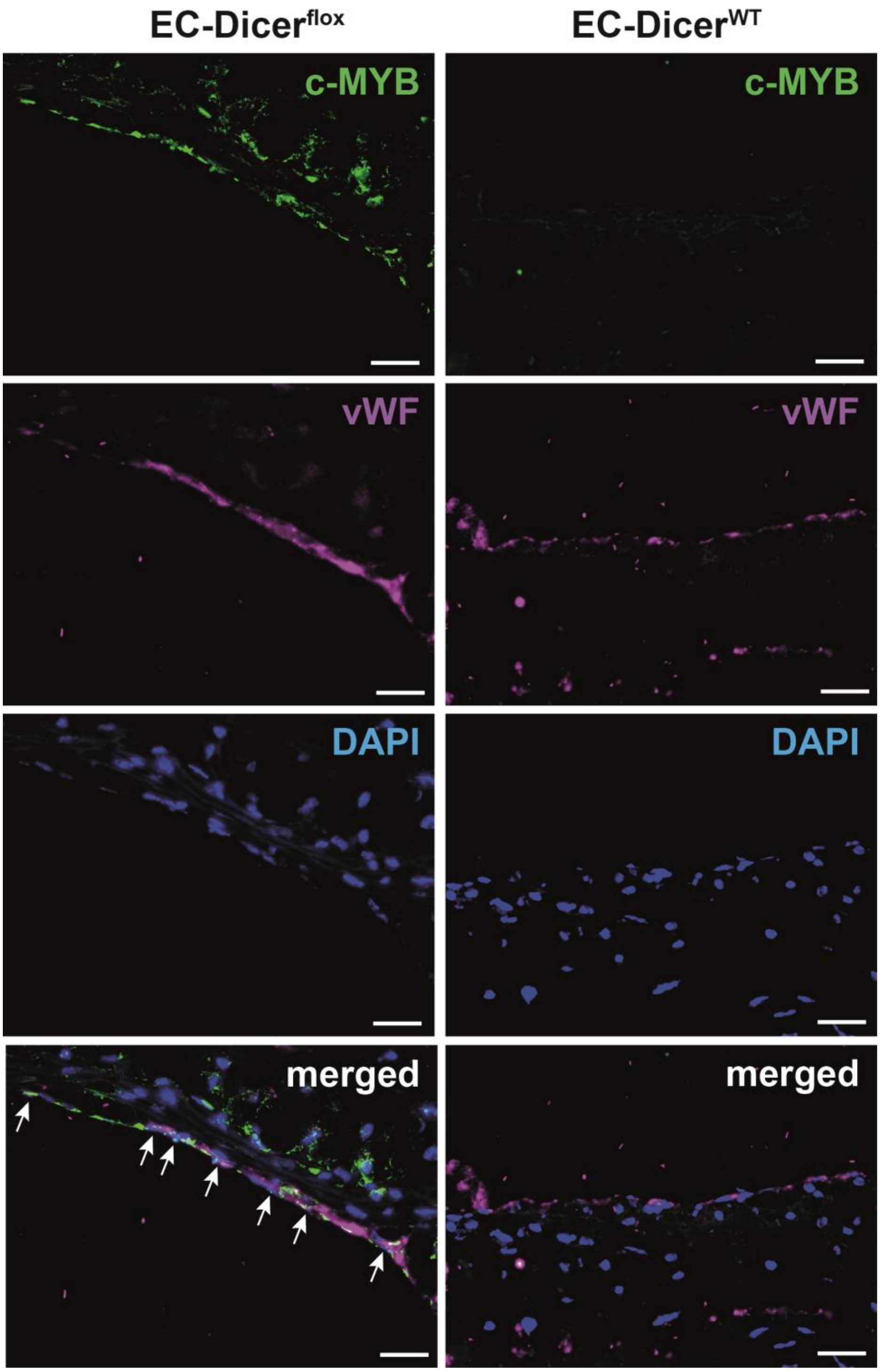

Supplementary Figure 9. c-Myb expression in ECs. Immunostaining of c-MYB and von Willebrand factor (vWF) in aortic root sections after 12 weeks of HFD feeding. Arrows indicate c-MYB expressing ECs. The nuclei were stained with DAPI. Scale bar, 25 µm.

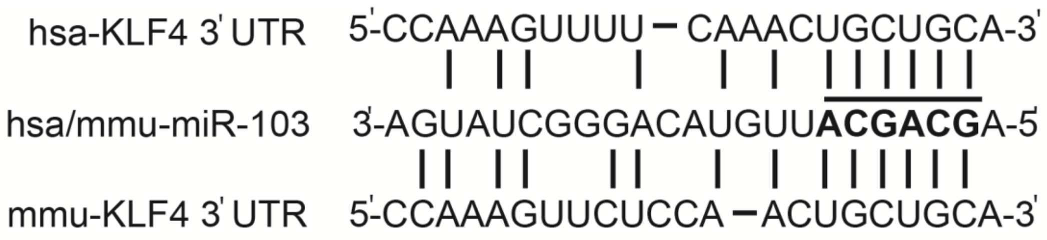

Supplementary Figure 10. Target site for human and murine (hsa/mmu) miR-103 in the 3′ untranslated region (UTR) of *KLF4* mRNA[1]. The seed sequence of miR-103 is highlighted in bold.

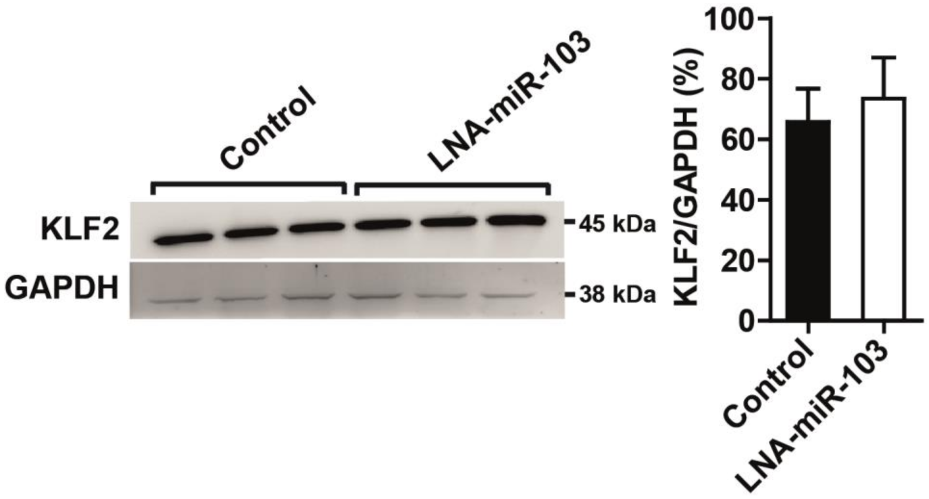

Supplementary Figure 11. Effects of miR-103 on KLF2 protein expression. Western blot analyses of KLF2 in HAECs treated with LNA-inhibitors of miR-103 or non-targeting control oligonucleotides. The expression levels were normalized to those of GAPDH (n = 3 per group). Full scans of Western Blots are provided in Supplementary Figure 15. The data are represented as the mean ± s.e.m. of the indicated number (n) of repeats. Student *t*-test was used to compare the groups.

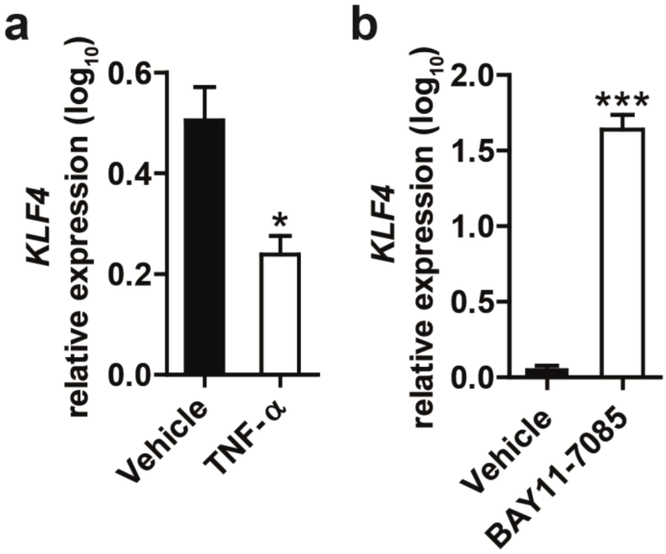

Supplementary Figure 12. NF-κB reduces *KLF4* expression in ECs. (a) Quantitative RT-PCR analyses of *KLF4* mRNA expression in HAECs with TNF-α stimulation (n = 4 per group). (b) *KLF4* mRNA expression in HAECs treated with vehicle or the NF-κB-inhibitor BAY11-7085 (n = 5 per group). The data are represented as the mean ± s.e.m. of the indicated number (n) of repeats. $*P < 0.05$ and $***P < 0.001$. Student *t*-test was used to compare the groups.

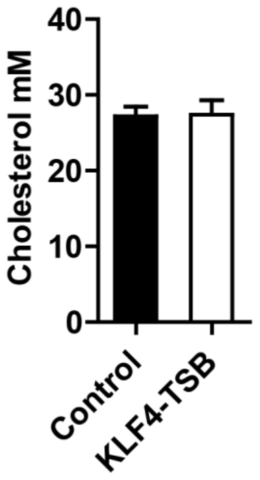

Supplementary Figure 13. Effect of KLF4-Target Site Blockers (KLF4-TSBs) treatment on serum cholesterol levels. Cholesterol levels were analyzed in the serum of Apoe$^{-/-}$ mice treated with KLF4-TSBs or control oligonucleotides after 8 weeks HFD feeding (n = 6–7 mice per group). The data are represented as the mean ± s.e.m. of the indicated number (n) of repeats. Student *t*-test was used to compare the groups.

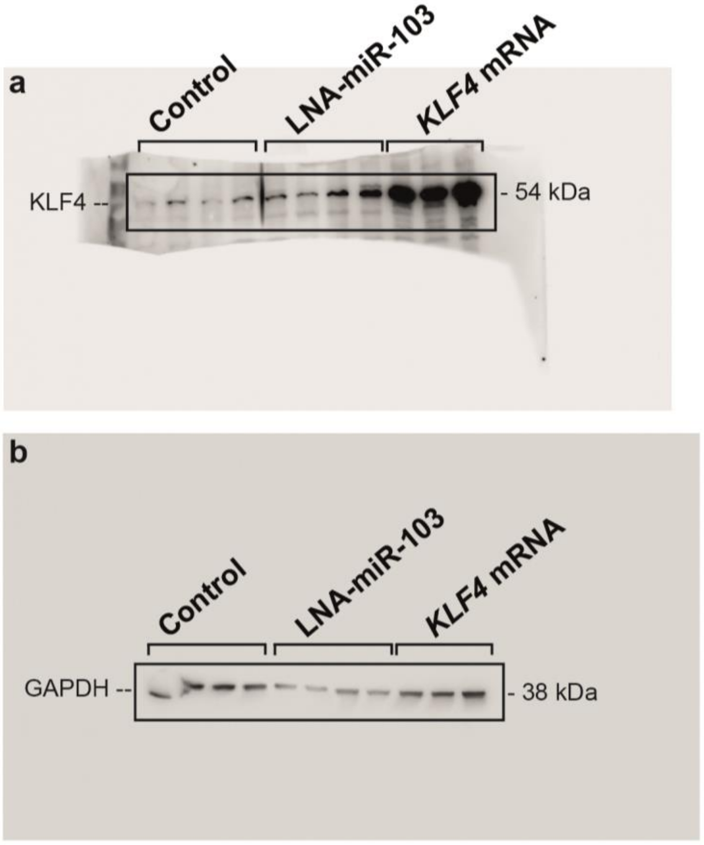

Supplementary Figure 14. Full scans of the Western Blots for KLF4 and GAPDH.

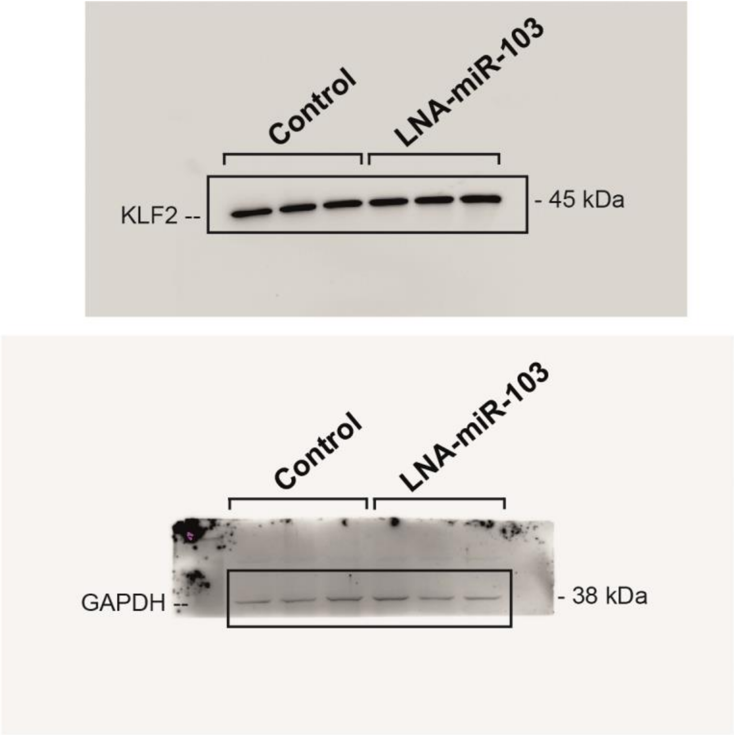

Supplementary Figure 15. Full scans of the Western Blots for KLF2 and GAPDH.

Supplementary Tables

Supplementary Table 1. Significantly downregulated miRNAs in EC-Dicer^{flox} compared with EC-Dicer^{WT} mice after 4 weeks of HFD feeding (n = 3 mice per group).

miRNAs	log_{10} RQ	neg log_{10} *P*-value
miR-465C-5p	−0.52	2.17
miR-142-5p	−0.34	1.64
miR-433	−0.27	1.34
miR-495	−0.20	2.17
miR-331-3p	−0.18	2.35
miR-301b	−0.18	3.29
miR-301a	−0.18	2.59
miR-615-3p	−0.18	1.35
miR-487b	−0.17	1.77
miR-425	−0.13	1.53
miR-652	−0.11	1.65
miR-18a*	−0.11	1.38
miR-103	−0.10	3.50
miR-1191	−0.09	1.51

Supplementary Table 2. Significantly downregulated miRNAs in EC-Dicer^{flox} compared with EC-Dicer^{WT} mice after 12 weeks of HFD feeding (n = 3 mice per group).

miRNAs	$\log_{10}$ RQ	neg $\log_{10}$ *P*-value
miR-183	−0.57	1.60
miR-433	−0.50	2.35
let-7d*	−0.35	1.32
miR-669D	−0.21	1.51
miR-1198	−0.21	1.89
miR-667	−0.19	1.68
miR-103	−0.17	1.50
miR-106b*	−0.16	2.52
miR-301b	−0.16	1.50
miR-99b	−0.15	3.36
let-7i	−0.14	2.16
miR-324-3p	−0.13	1.71
miR-652	−0.13	1.35
miR-136	−0.13	1.96
miR-384-3p	−0.11	1.48
miR-434-3p	−0.07	1.34
let-7b	−0.04	2.34
miR-872*	−0.02	1.30

Supplementary Table 3. PCR primer sequences

Gene	Primer Sequence
Human:	
CX_3CL1	sense 5'-GCAAACGCGCAATCATCTTG-3'
	antisense 5'-TTGACCCATTGCTCCTTCGG-3'
CXCL1	sense 5'-CCCAAACCGAAGTCATAGCCA-3'
	antisense 5'-GATGCAGGATTGAGGCAAGC-3'
CCL2	sense 5'-CGCTCAGCCAGATGCAATCAA-3'
	antisense 5'-GACACTTGCTGCTGGTGATTC-3'
KLF4	sense 5'-ATCTCAAGGCACACCTGCG-3'
	antisense 5'-CCTGGTCAGTTCATCTGAGCG-3'
GAPDH	sense 5'-AGGGCTGCTTTTAACTCTGGT-3'
	antisense 5'-CCCCACTTGATTTTGGAGGGA-3'
c-MYB	sense 5'-AAGCTACTGCCTGGACGAAC-3'
	antisense 5'-TTCCTGTTCGACCTTCCGAC-3'
TCF7	sense 5'-TGCTCCAAGTGGTGGGAATC-3'
	antisense 5'-GCAGGCAGCCATAGGTACAA-3'
Mouse:	
Dicer1	sense 5'-GAATAAGGCTTATCTTCTGCAGG-3'
	antisense 5'-CATAAAGGTGCTTGGTTATGAGG-3'
Klf4	sense 5'-GACTAACCGTTGGCGTGAGG-3'
	antisense 5'-CGGGTTGTTACTGCTGCAAG-3'
c-Myb	sense 5'-CGTACCTAAGAACAGGCCCC-3'
	antisense 5'-GCGTTCACGTATTTCCGAGC-3'
Tcf7	sense 5'-AGGTGGCATGCACTATCTCG-3'
	antisense 5'-TTTCCCTTGACCGCCTCTTC-3'
Lef1	sense 5'-GATCCTGGGCAGAACATGGC-3'
	antisense 5'-GCTGTCTCTCTTTCCGTGCT-3'
Dkk2	sense 5'-TCTAGGAAGGCCACACTCCA-3'
	antisense 5'-TGGGTCTCCTTCATGTCCTTT-3'
Sox17	sense 5'-TTCCATCTCCACCTCCGACC-3'
	antisense 5'-GTCGATTGGCACCTTTCACC-3'
B2m	sense 5'-TCGGTGACCCTGGTCTTTCT-3'
	antisense 5'-TTTGAGGGGTTTTCTGGATAGCA-3'
Mouse/Human:	
Taq-in situ-miR-103 RT	5'-GTCGTATCCAGTGCAGGGTCCGAGGTAT TCGCACTGGATACGACTCATA-3'
Taq-in situ-miR-103	sense 5'-GCCCAGCAGCATTGTACAG-3'
	antisense 5'-GTGCAGGGTCCGAGGT-3'
C. elegans:	
Taq-in situ-cel-miR-39 RT	5'-GTCGTATCCAGTGCAGGGTCCGAGGTAT TCGCACTGGATACGACCAAGC-3'
Taq-in situ-cel-miR-39	sense 5'-GCCCTCACCGGGTGTAAAT-3'
	antisense 5'-GTGCAGGGTCCGAGGT-3'

Supplementary Reference

1 Chen, H. Y. *et al.* miR-103/107 promote metastasis of colorectal cancer by targeting the metastasis suppressors DAPK and KLF4. *Cancer research* **72**, 3631-3641, doi:10.1158/0008-5472.CAN-12-0667 (2012).
